# A Structure-Adaptive Hybrid RBF-BP Classifier with an Optimized Learning Strategy

**DOI:** 10.1371/journal.pone.0164719

**Published:** 2016-10-28

**Authors:** Hui Wen, Weixin Xie, Jihong Pei

**Affiliations:** ATR Key Lab of National Defense, shenzhen University, shenzhen 518060, China; College of Bioinformatics Science and Technology, CHINA

## Abstract

This paper presents a structure-adaptive hybrid RBF-BP (SAHRBF-BP) classifier with an optimized learning strategy. SAHRBF-BP is composed of a structure-adaptive RBF network and a BP network of cascade, where the number of RBF hidden nodes is adjusted adaptively according to the distribution of sample space, the adaptive RBF network is used for nonlinear kernel mapping and the BP network is used for nonlinear classification. The optimized learning strategy is as follows: firstly, a potential function is introduced into training sample space to adaptively determine the number of initial RBF hidden nodes and node parameters, and a form of heterogeneous samples repulsive force is designed to further optimize each generated RBF hidden node parameters, the optimized structure-adaptive RBF network is used for adaptively nonlinear mapping the sample space; then, according to the number of adaptively generated RBF hidden nodes, the number of subsequent BP input nodes can be determined, and the overall SAHRBF-BP classifier is built up; finally, different training sample sets are used to train the BP network parameters in SAHRBF-BP. Compared with other algorithms applied to different data sets, experiments show the superiority of SAHRBF-BP. Especially on most low dimensional and large number of data sets, the classification performance of SAHRBF-BP outperforms other training SLFNs algorithms.

## Introduction

In the field of pattern recognition and data mining, as typical single-layer feed-forward networks (SLFNs), radial basis function networks (RBF) have been intensively studied over the past several decades. When used for classifying problems, there are three important factors for evaluating network performance: 1) classifying accuracy, 2) network size, and 3) training time. To achieve good network performance, different optimization algorithms are used to train the RBF hidden layer, such as K-means clustering [1, 2], fuzzy C-means clustering [3, 4], fuzzy K-nearest neighbors [5], differential evolution [6, 7], and other optimization algorithms [8–12]. However, in most of these methods, the number of RBF hidden nodes is assigned a priori, which may lead to poor adaptability for different sample sets. The selection of network size is also a critical issue. If there are too few hidden nodes the network may not be able to approximate the given function, and if there are too many, the network may exhibit poor generalization performance because of over fitting. Several sequential learning algorithms have been proposed to find a proper network size [13–16]. In [17], a minimal resource allocation network (MRAN) is proposed, which is allowed to delete the previous center. The deletion strategy is based on the overall contribution of each hidden unit to the network output. A sequential learning algorithm for growing and pruning the RBF (GAP-RBF) and a generalized growing and pruning RBF (GGAP-RBF) algorithm are proposed in [18, 19], which use the significance of nodes as the learning strategy. Because the GGAP-RBF algorithm could not handle problems with high-dimensional probability density distribution, this problem is overcome in [20], which uses a Gaussian mixture model (GMM) to approximate the GGAP (GGAP-GMM) evaluation formula. In [21], an error correction (ErrCor) algorithm is used for function approximation. In each iteration of the algorithm, one RBF unit is added to fit and then eliminate the highest peak in the error surface, which can reach a desired error level with fewer RBF units. Other methods have also been established to identify a proper structure while maintaining the desired level of accuracy [22–27].

For online training algorithms, the training time is very important. This parameter directly determines how efficiently an algorithm runs. This problem is well overcome by extreme learning machines (ELMs) [28], which are also effective algorithms for training SLFNs; ELMs choose random hidden node parameters and calculate the output weights with the least squares algorithm. This method can achieve a fast training speed, as well as good classifying accuracy. In ELMs, the number of hidden nodes is assigned a priori, and many non-optimal nodes may exist; ELM tends to require more hidden nodes than conventional tuning-based algorithms [29]. Thus, in [30–33], several types of growing and pruning techniques based on ELMs are proposed to effectively estimate the number of hidden nodes. In [34], an evolutionary ELM (E-ELM) based on differential evolution and ELM is proposed; the algorithm uses the differential evolution method to optimize the network input parameters and an ELM algorithm to calculate the network output weights. Because the trial vector generation strategies and the control parameters have to be manually chosen in E-ELM, in [35], a self-adaptive evolutionary extreme learning machine (SaE-ELM) is proposed; its network hidden node parameters are optimized by the self-adaptive differential evolution algorithm, which further improves the network performance.

This paper mainly focuses on how to obtain higher classifying accuracy as well as a suitable network size for the RBF hidden layer. A structure-adaptive hybrid RBF-BP (SAHRBF-BP) classifier with an optimized learning strategy is presented. SAHRBF-BP is composed of a structure-adaptive RBF network and a BP network of cascade, where the number of RBF hidden nodes is adjusted adaptively according to the distribution of sample space, and a suitable network size for RBF hidden layer that matches the complexity of the sample space can be built up. Thus, SAHRBF-BP solves the problem of dimension change from sample space mapping to feature space. In SAHRBF-BP, the nodes in the RBF network are used for nonlinear kernel mapping, the complexity of sample space is mapped onto the dimension of the BP input layer, and the BP network is then used for nonlinear classification. The nonlinear kernel mapping can improve the separability of sample spaces, and a nonlinear BP classifier can then supply a better classification surface. In this manner, SAHRBF-BP combines the local response characteristics of the RBF network with the global response characteristics of the BP network, which simplifies the selection of parameters in the BP hidden layer while reducing the dependence on space mapping in the RBF hidden layer; thus, the classification accuracy is improved while the generalization performance is guaranteed.

An optimized learning strategy is presented to construct SAHRBF-BP classifier. The optimized learning strategy uses global information of training sample space and generates RBF hidden nodes incrementally. On the one hand, many optimization algorithms, such as K-means clustering, fuzzy C-means clustering, differential evolution, also use global information of training sample space to optimize RBF hidden nodes, however, the number of hidden nodes in these optimization algorithms needs to be manually determined, which may lead to poor adaptivity for different sample sets. On the other hand, the sequential learning algorithms, such as MRAN, GAP-RBF, can achieve the estimation of RBF hidden nodes for different sample sets, however, the loss of global information may lead to a reduction in classification performance. In addition, unlike GAP-RBF, the presented method does not require an assumption that the input samples obey a unified distribution. Unlike GGAP-GMM, it does not need to fit the input sample distribution. By using a potential function clustering approach to measure the density in each class of training sample space, the corresponding RBF hidden nodes that cover different sample regions can be established. It reduces the restrictions on the sample sets and is adaptable to more complex sample sets. Once an initial RBF hidden node is generated, a form of heterogeneous samples repulsive force is designed to further optimize the hidden node parameters. For each initial hidden node, in a certain region, we assume the heterogeneous samples can affect the center; that is, there is an repulsive force that makes the current center move away from the heterogeneous samples. When the center reaches a suitable position, the repulsive force will disappear. Then, a suitable width parameter can be determined accordingly. A mechanism for eliminating the potentials of the original samples is then presented. This mechanism is ready for the next learning step. Thus, the RBF centers and the width and number of RBF hidden nodes can be effectively estimated.

Once the number of RBF hidden nodes is generated adaptively and the node parameters are optimized, the number of subsequent BP input nodes can be determined, and the overall SAHRBF-BP classifier is built up; then different training sample sets are used to train the BP network parameters in SAHRBF-BP, where the BP network parameters are optimized by the existing BP algorithm.

In this paper, the performance of SAHRBF-BP is compared with that of other well-known training SLFNs algorithms, such as back propagation based on stochastic gradient descent(SGBP) [36], MRAN, SVM, ELM, and SaE-ELM on 108 benchmark data sets. To measure the unique features of SAHRBF-BP, the RBF nerwork based on k-means clustering (KMRBF) [2], GAP-RBF and the k-means clustering learning algorithm based on the hybrid RBF-BP network (KMRBF-BP) are added to compare with SAHRBF-BP on two artificial data sets. Experiments show that the superiority of SAHRBF-BP. Especially on most low dimensional and large number of data sets, the classification performance of SAHRBF-BP outperforms other training SLFNs algorithms.

## Methods

### SAHRBF-BP classifier

**Fig 1:**
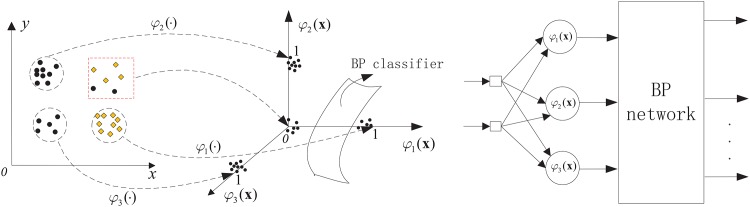
Illustrative diagram of the sample space mapping onto feature space for different sample sets. (A) The mapping dimension is 3 (B) The mapping dimension is 6.

SAHRBF-BP is composed of a structure-adaptive RBF network and a BP network of cascade, where the number of RBF hidden nodes are adjusted adaptively according to the distribution of sample space, the complexity of sample space is mapped onto the dimension of the BP input layer, and the BP network is then used for nonlinear classification. The nonlinear kernel mapping can improve the separability of sample space, and a nonlinear BP classifier can then supply a better classification surface. To clarify the situation, Fig 1(A) and 1(B) show an illustrative diagram of sample space mapping onto feature space for different classification problems. Note that in Fig 1(A), the samples in the red box far away from the center of each kernel function will be mapped near the origin of the coordinate plane, this problem will be overcome by the optimized learning strategy presented in the next section. In Fig 1(B), with the increase in sample space complexity, the dimension of the feature space is increased accordingly, which is classified by a BP network.

**Fig 2:**
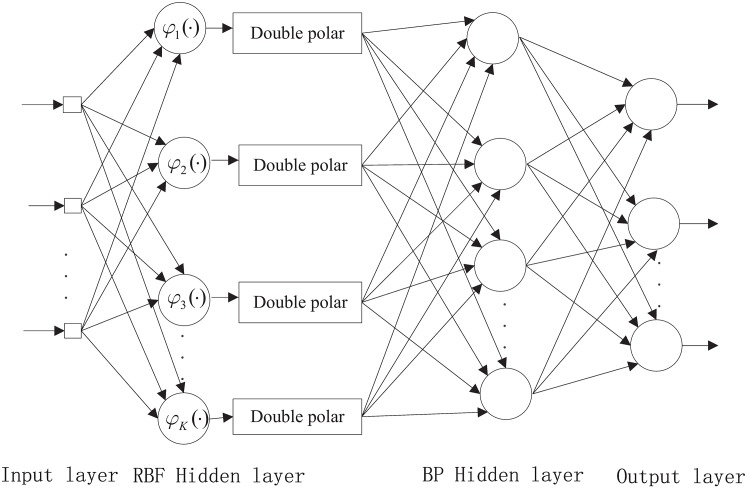
SAHRBF-BP classifier.

SAHRBF-BP is shown in Fig 2, which consists of four components:

1. The input layer, which consists of *t* source neurons, where *t* is the dimensionality of the input vector.
2. The RBF hidden layer, which consists of a group of Gaussian kernel functions:
  $$\begin{array}{c} \varphi_{k}\left(x\right)=\exp\left(-\frac{1}{2\sigma_{k}^{2}}{||x-\mu_{k}\left|\right|}^{2}\right),\;k=1,2,...K \end{array}$$ (1)
  where *μ*_*k*_ and *σ*_*k*_ are the center and width of the hidden node, respectively, and *K* is the number of hidden neurons.
3. The BP hidden layer, which consists of the neurons between the RBF hidden layer and output layer. The induced local field $v_{j}^{\left(l\right)}$ for node *j* in layer *l* of the BP is
  $$\begin{array}{c} v_{j}^{\left(l\right)}=\sum_{i}\omega_{ji}^{\left(l\right)}y_{i}^{\left(l-1\right)} \end{array}$$ (2)
  where $y_{i}^{\left(l-1\right)}$ is the output signal of the neuron *i* in the previous layer *l* − 1 of the BP network and $\omega_{ji}^{\left(l\right)}$ is the synaptic weight of neuron *j* in layer *l* that is fed from neuron *i* in layer *l* − 1. Assuming the use of a sigmoid function, the output signal of neuron *j* in layer *l* is
  $$\begin{array}{c} y_{j}^{\left(l\right)}=\varphi_{j}\left(v_{j}\right)=a\tanh\left(bv_{j}\right) \end{array}$$ (3)
  where *a* and *b* are constants.
  If neuron *j* is in the first BP network hidden layer, i.e., *l* − 1, set
  $$\begin{array}{c} y_{j}^{\left(0\right)}=g_{j}\left(x\right) \end{array}$$ (4)
  where *g*_*j*_(**x**) is the double polar output of *φ*_*j*_(**x**) and can be denoted as
  $$\begin{array}{c} g_{j}\left(x\right)=2\cdot \varphi_{j}\left(x\right)-1 \end{array}$$ (5)
4. The output layer. Set *L* is the depth of the BP network, note the depth of the BP network is equal to the sum of the BP network input layer, the hidden layer, and the output layer, i.e., if *l* = 1, then *L* = 3, and the output can be given as
  $$\begin{array}{c} o_{j}=y_{j}^{\left(L\right)} \end{array}$$ (6)

In Fig 2, the double polar processing can ensure the validity of the BP network input. In addition, the combination of the structure-adaptive adjustment of the RBF hidden layer with the BP network can provide a good complementary effect. On the one hand, the RBF network has good stability, where the activation response in the RBF hidden nodes has local characteristics and maps the output to a value between 0 and 1. Thus, the original samples, including outliers, will be limited to a finite space, and the adaptive adjustment of RBF hidden nodes can ensure the validity of the space mapping. Processing the results of mapping the RBF hidden nodes and using them for the input of the BP network can reduce the dependence on the selection of BP network parameters; furthermore, the convergence rate of the BP network can be increased and local minima can be avoided. On the other hand, in a BP network, the activation response in hidden nodes has global characteristics, especially those regions not fully displayed in the training set. In SAHRBF-BP, the BP network is used for nonlinear classification, which can reduce the dependence on the original sample space mapping. Even if there are errors in the original sample space mapping, the nonlinear BP network can be compensated for to a certain extent. Therefore, SAHRBF-BP combines the stability of the RBF network with the generalization ability of the BP network and improves the classification performance further.

A single hidden layer multilayer perceptron neural network with input-output mapping can provide an approximate realization of any continuous mapping [37]. In light of the foregoing discussion, in SAHRBF-BP, we set the number of BP hidden layers to *l* = 1, and the number of hidden nodes of BP should be appropriately increased with the increase in the complexity of the sample space.

When SAHRBF-BP classifier is built up, however, new problems may arise because the number of RBF hidden nodes and their parameters are unknown, and inappropriate kernel mapping will deteriorate the network performance. This process can be overcome by the optimized learning strategy presented in the next section.

### The optimized learning strategy

#### Main objective

**Fig 3:**
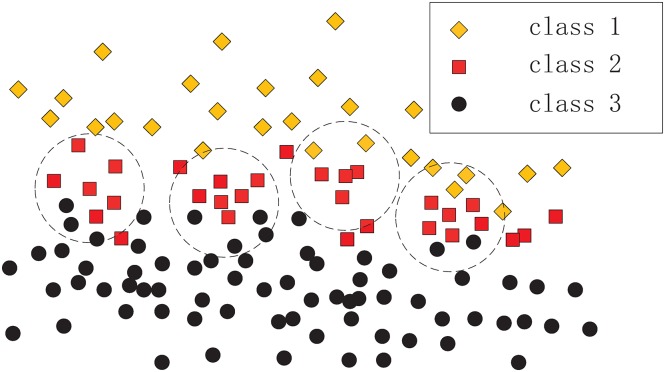
Example of the current RBF hidden nodes covering other classes of samples.

To obtain good classifying performance for a given training sample set, it is necessary to fully use training sample information. Fig 3 demonstrates such a scenario, where the generated RBF hidden nodes are used to cover samples of class 2. However, these RBF hidden nodes may cover samples of classes 1 and 3, which leads to a reduction in classification performance. Our main objective is to design a method that can optimize the coverage of each class of training samples, where each coverage generates a RBF hidden node and ultimately estimates the center, the width and the number of RBF hidden nodes. For that purpose, the following issues should be considered:

1) To optimize the coverage of the training sample space, a suitable initial RBF hidden node must be established each time.
2) The adjustments of the center and width should meet certain criteria such that each generated RBF hidden node can cover the samples of the current class as much as possible, while covering the samples of other classes as little as possible.

To address issue 1), we consider that for each class of training samples, in different regions, their densities are different. To cover the training sample space effectively, the sample in the most intensive region can be selected as the initial center. Therefore, it is necessary to quantify each class of samples and establish a standard for measuring the density of the input sample space. In this paper, a potential function is introduced into training sample space. By calculating the sample potentials in each class, the densities of different regions can be measured, where the sample with the maximum potential value can be used as the initial center. To address issue 2), we consider that the information of other classes of samples can be used to adjust the center and width such that an optimization model is established, where a form of heterogeneous samples repulsive force is designed to adjust the center and the width adaptively.

To complete the main objective, the following steps can be followed.

Step 1 Compute the potential value of each sample in the current class.
Step 2 Set the sample with the maximum potential value as the initial center.
Step 3 Consider the distance between the heterogeneous samples and the center; in a certain region, the center and width should be adjusted adaptively by a form of heterogeneous samples repulsive force.
Step 4 Eliminate the potential value of each sample in the current class.
Step 5 Iterate Steps 2-4 until the stop condition is met, then turn to learn other classes of samples.

#### Algorithm principle

In the field of pattern recognition, potential functions can be used for density clustering and image segmentation (IS). Several methods of constructing potential function are proposed in [38]; here, we choose the potential function

$$\begin{array}{c} \gamma \left(x_{1},x_{2}\right)=\frac{1}{1+T\cdot d^{{}^{2}}\left(x_{1},x_{2}\right)} \end{array}$$ (7)

where *γ*(**x**_**1**_, **x**_**2**_) represents the interaction potential of two points **x**_**1**_, **x**_**2**_ in the input sample space, *d*(**x**_**1**_, **x**_**2**_) represents the distance measure, and *T* is a constant, which can be regarded as the distance weighting factor.

Given a training sample set *S*, where a specific label **y**_**i**_, **y**_**i**_ ∈ {**y**_**i**_; *i* = 1, 2, … *h*} is attached to each sample vector **x** in *S*, *h* is the number of pattern class. Let *S*_*i*_ denote the set of feature vectors that are labeled **y**_**i**_, $S_{i}=\left\{x_{1}^{i},x_{2}^{i},...,x_{N_{i}}^{i}\right\}$, where *N*_*i*_ is the number of training samples in the *ith* pattern class. Thus $S=\cup_{i=1}^{h}S_{i}$, *S*_*i*_ ∩ *S*_*j*_ = ∅, ∀*i* ≠ *j*. For a pair of samples $\left(x_{u}^{i},x_{v}^{i}\right)$ in *S*_*i*_, its interaction potential can be denoted as

$$\begin{array}{c} \gamma \left(x_{u}^{i},x_{v}^{i}\right)=\frac{1}{1+T\cdot d^{{}^{2}}\left(x_{u}^{i},x_{v}^{i}\right)} \end{array}$$ (8)

Let $x_{v}^{i}$ be the baseline sample; therefore, the interaction potential of all other samples to $x_{v}^{i}$ can be denoted as

$$\begin{array}{c} \rho \left(x_{v}^{i}\right)=\sum_{u=1,u\neq v}^{N_{i}}\gamma (x_{u}^{i},x_{v}^{i}) \end{array}$$ (9)

Once the potentials of each sample in *S*_*i*_ are given, the sample with the maximum potential can be selected, where it is assumed the sample is $x_{p}^{i}$, that is,

$$\begin{array}{c} \rho \left(x_{p}^{i}\right)=\max\left\{\rho \left(x_{1}^{i}\right),\rho \left(x_{2}^{i}\right),...,\rho \left(x_{N_{i}}^{i}\right)\right\} \end{array}$$ (10)

To generate valid Gaussian kernel functions, we find the densest region in the sample space and then establish a Gaussian kernel to cover the region. To that end, the sample with the maximum potential is chosen as the initial center of the Gauss kernel function, which is expressed as follows:

$$\begin{array}{c} \mu_{k}=x_{p}^{i} \end{array}$$ (11)

where *k* refers to the number of RBF hidden neurons generated.

Once the width is given, an initial RBF hidden node is established, which can be used to cover samples of the current class. However, the generated RBF node takes into account sample information about the current class only, which may cause the current RBF hidden node to cover samples of other classes. To achieve the optimization coverage of each class of training samples, here, heterogeneous samples are taken into account to optimize the initial hidden node parameters. A form of heterogeneous samples repulsive force is used to adjust the center and the width adaptively. To make the center adjustment, first, the direction of each heterogeneous sample repulsive force should be in line with the centerline, which can make the center far away from the heterogeneous sample directly. Second, when a heterogeneous sample is close to the center, the magnitude of the center should be adjusted by a large margin, whereas when a heterogeneous sample is relatively far from the center, the magnitude of the center should be adjusted slightly. According to the foregoing description, the heterogeneous sample repulsive force is defined as follows:

**Definition**: Given two vectors, where one is the center and the other is a heterogeneous sample, there is a repulsive force that makes the heterogeneous sample point to the center. The magnitude of the force is inversely proportional to the square of the distance between the two vectors, and the direction of the repulsive force is in line with the centerline.

To adjust the center by the form of heterogeneous sample repulsive force, two hypothetical conditions should be met:

1) When the initial center is determined, given the initial width, only in the current coverage region, the heterogeneous sample repulsive force exists.
2) When the center is adjusted to a suitable position, the heterogeneous sample repulsive force will disappear.

Condition 1) demonstrates the case when the distance between the center and the heterogeneous samples is outside a certain range, the heterogeneous sample repulsive force can be ignored; this condition simplifies the study of the problem. For condition 2), the key is to establish criteria to make the center converge toward a suitable position.

According to the definition of the heterogeneous sample repulsive force and condition 1), the optimization model can be given as follows.

Given the initial width *σ*, when the initial center ***μ***_**k**_ is generated, where $\mu_{k}{=x}_{p}^{i}\in S_{i}$. For a heterogeneous sample $x_{q}^{j}$, $x_{q}^{j}\notin S_{i}$, if $\left|\right|x_{q}^{j}-\mu_{k}\left|\right|<\lambda \cdot \sigma$, where *λ* is a width covering factor, there is a repulsive force from $x_{q}^{j}$ to ***μ***_**k**_, which can be denoted as

$$\begin{array}{c} F_{x_{q}^{j}}\propto \frac{1}{d(x_{q}^{j},\mu_{k})}\cdot \frac{x_{q}^{j}-\mu_{k}}{\left|\right|x_{q}^{j}-\mu_{k}\left|\right|} \end{array}$$ (12)

Here, a negative exponential function is chosen to express the relationship between the heterogeneous sample repulsive force and the distance:

$$\begin{array}{c} F_{x_{q}^{j}}=\exp\left(-\alpha \cdot d\left(x_{q}^{j},\mu_{k}\right)\right)\cdot \frac{x_{q}^{j}-\mu_{k}}{\left|\right|x_{q}^{j}-\mu_{k}\left|\right|} \end{array}$$ (13)

where *α* is a positive constant and can be seen as the heterogeneous sample repulsive force control factor. Assume the number of heterogeneous samples in the current covered region is *M*_*j*_. Adding all the heterogeneous samples repulsive force, the center can be adjusted as follows:

$$\begin{array}{c} \mu_{k}^{\prime }=\mu_{k}+\sum_{q=1}^{M_{j}}F_{x_{q}^{j}} \end{array}$$ (14)

Note that for a sample **x**, when **x** ∉ *S*_*i*_, if |**x** − ***μ***_**k**_|| < *λ* ⋅ *σ*, *M*_*j*_ counts plus 1. Similarly, set *M*_*i*_ denotes the number of samples in the current class in the current covered region, when **x** ∈ *S*_*i*_, if ||**x** − ***μ***_**k**_|| < *λ* ⋅ *σ*, *M*_*i*_ counts plus 1.

**Fig 4:**
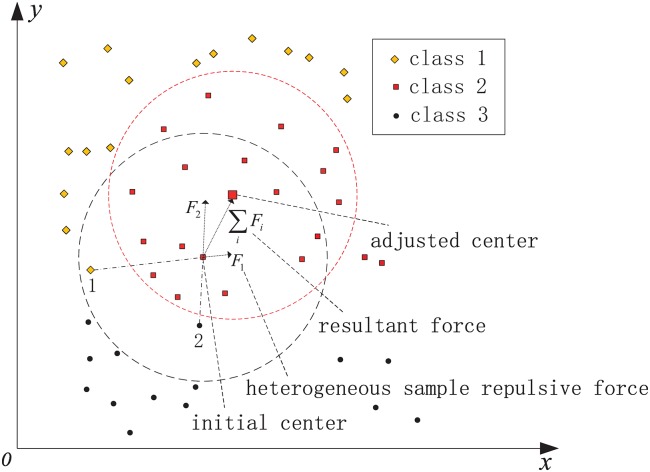
Model of heterogeneous sample repulsive force.

Fig 4 provides a geometrical description of the heterogeneous sample repulsive force model, where the black and red boxes denote the covered region before and after the center adjustment, respectively. In Fig 4, there is a repulsive force makes samples 1 and 2 point to the initial center. The resultant force adjusts the initial center to a new position that is far away from samples 1 and 2.

For condition 2), to make the center reach a suitable position, it is necessary to carry out multiple iterations. Set *M* is the iteration step variable, at the initial stage, the magnitude of the center adjustment is relatively large; with the increase in the iteration step, the magnitude of the center adjustment will gradually decrease and eventually converge to a suitable position. To ensure validity of the center adjustment, set $M_{i}^{\prime }$ and $M_{j}^{\prime }$ represent the number of current class of samples and heterogeneous samples covered in the updated region, respectively, for each center adjustment, Eq (14) can be corrected as follows:

$$\begin{array}{ccc} \mu_{k}^{\prime } & = & \mu_{k}+\frac{1}{M}\sum_{q=1}^{M_{j}}F_{x_{q}^{j}}\\ s.t. & & M_{i}^{\prime }\geq M_{i}\;\text{and}\;M_{j}^{\prime }\leq M_{j} \end{array}$$ (15)

In practice, because of the complexity of different training sample sets, even if the center is adjusted to a suitable position, the generated RBF hidden nodes may also cover heterogeneous samples under the given initial width parameters. Decreasing the width may be one way of reducing the coverage of heterogeneous samples; however, if the width is too small, the generalization performance will be greatly reduced. To further complete the optimization coverage of different regions of the each class of training samples, and guarantee good generalization performance, the width adjustment is set as follows:

$$\begin{array}{c} \sigma_{k}=\{\begin{array}{cc} \max\{\min\left(d\left(\mu_{m}^{\prime },x_{q}^{j}\right)/\beta \right),\sigma_{\min}\}, & \text{if}\;M_{j}^{\prime }>0.\\ \sigma , & \text{if}\;M_{j}^{\prime }=0. \end{array} \end{array}$$ (16)

where *σ*_min_ is the minimum width, *β* is a fixed value and can be seen as the width constraint factor. In practice, to ensure validity of the width adjustment, the selection of *β* should be a little less than the width covering factor *λ* and the adjusted width should be in the range between *σ*_min_ and *σ*. For different RBF hidden nodes, this adjustment can ensure the relative difference of the widths to better fit the training sample space. Note that the adjustment of the width is carried out only once, after the center adjustment.

When a hidden node is established, it is necessary to eliminate the potentials of the region to find the next initial center in the remaining samples. This process can be accomplished as follows:

$$\begin{array}{c} \rho^{\prime }\left(x_{n}^{i}\right)=\rho \left(x_{n}^{i}\right)-\rho \left(x_{p}^{i}\right)\cdot \exp\left(-\frac{1}{2\sigma_{k}^{2}}{\left|\right|x_{n}^{i}-x_{p}^{i}\left|\right|}^{2}\right),\;n=1,2,...N_{i} \end{array}$$ (17)

where $x_{p}^{i}$ is the initial center of the current hidden neuron. For the potential value update process, Eq (17) shows when a sample $x_{n}^{i}$ is close to the initial center $x_{p}^{i}$, the potential value of $x_{n}^{i}$ is attenuated fast, whereas when a sample $x_{n}^{i}$ is far away from the center, the potential value of $x_{n}^{i}$ is attenuated slowly. When meeting the inequality

$$\begin{array}{c} \max\{\rho^{\prime }\left(x_{1}^{i}\right),\rho^{\prime }\left(x_{2}^{i}\right),...,\rho^{\prime }\left(x_{N_{i}}^{i}\right)\}>\delta \end{array}$$ (18)

the learning process goes on and is ready to search the next initial center. Otherwise, the algorithm of constructing RBF hidden nodes in the current pattern class is terminated and turns to learn other pattern classes, where *δ* is a threshold.

**Fig 5:**
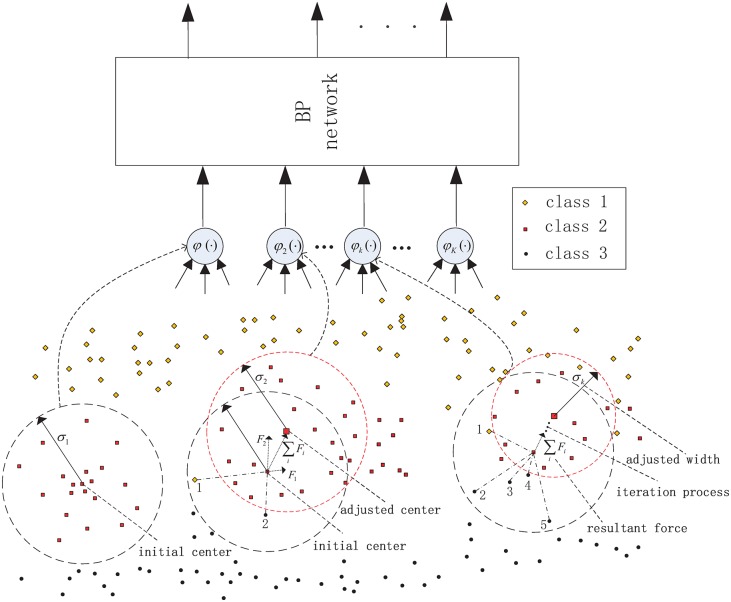
Illustrative diagram of adaptively generating RBF hidden nodes and optimizing node parameters.

Fig 5 shows the illustrative diagram of adaptively generating RBF hidden nodes and optimizing node parameters, where the number of RBF hidden nodes is increased incrementally, and each initial RBF hidden node is determined by a potential function clustering approach, then a form of heterogeneous sample repulsive force is used to further optimize each RBF hidden node parameters. In Fig 5, the black line box represents regions covered by the initial center and the width, the red line box represents the final coverage regions.

Combining SAHRBF-BP classifier, the optimized learning strategy is summarized in Algorithm 1.

Algorithm 1 The optimized learning strategy

 Initialization;

 *for* *i* = 1: *c*    % for each class of training samples

  Compute the potential value of each sample according to Eq (9).

  *while* $\max\{\rho \left(x_{1}^{i}\right),\rho \left(x_{2}^{i}\right),...,\rho \left(x_{N_{i}}^{i}\right)\}>\delta$

   Determine the maximum potential value of each sample according to Eq (10).

   The number of RBF hidden nodes counts plus 1, allocate an initial center using Eq (11).

   Determine *M*_*i*_, *M*_*j*_.

   Use Eqs (13) and (14) to update the initial center, determine $M_{i}^{\prime }$, $M_{j}^{\prime }$.

   *while* $M_{j}^{\prime }\neq 0\;\&\&\;M\leq \text{num\_Epoch}$

    *if* $M_{i}^{\prime }\geq M_{i}\;\&\&\;M_{j}^{\prime }\leq M_{j}$

$M_{i}\leftarrow M_{i}^{\prime }$, $M_{j}\leftarrow M_{j}^{\prime }$.

     Use Eq (15) to update the center, update $M_{i}^{\prime }$, $M_{j}^{\prime }$.

     *M* ← *M* + 1.

    *else*

     Use Eq (16) to update the width.

     Break;

    *end if*

   *end while*

   Eliminate the sample potential value of the region according to Eq (17).

  *end while*

 *end for*

 Use Eqs (1) and (5) to compute *g*_*j*_(**x**), let *g*(**x**) be the input of the BP network, where *g*(**x**) = (*g*_1_(**x**), *g*_2_(**x**), …, *g*_*K*_(**x**)).

 *while* ||**e**|| > mse_thres && *m* ≤ num_Epoch

  Use Eqs (2)–(4) and Eq (6) to compute the error signal *e*_*j*_ = *d*_*j*_ − *o*_*j*_, where *d*_*j*_ is the *jth* element of the desired response vector **d**.

  Compute the local gradients of the network as follow

$$\begin{array}{c} \delta_{j}^{\left(l\right)}=\left\{ \begin{array}{cc} e_{j}^{\left(L\right)}\phi_{j}^{\prime }\left(v_{j}^{\left(L\right)}\right) & \text{for neuron}\;j\;\text{in}\;\text{output}\;\text{layer}\;L\\ \phi_{j}^{\prime }\left(v_{j}^{\left(l\right)}\right)\sum_{k}\delta_{k}^{\left(l+1\right)}\omega_{kj}^{\left(l+1\right)} & \text{for neuron}\;j\;\text{in}\;\text{the}\;\text{BP}\;\text{hidden}\;\text{layer}\;l \end{array}\right. \end{array}$$

where $\phi_{j}^{\prime }\left(\cdot \right)$ is the differentiation with respect to the argument.

  Adjust the synaptic weights of the network in layer *l* of BP as below.

$$\begin{array}{c} \omega_{ji}^{\left(l\right)}\left(m+1\right)=\omega_{ji}^{\left(l\right)}\left(m\right)+\tau \left[\omega_{ji}^{\left(l\right)}\left(m-1\right)\right]+\eta \delta_{j}^{\left(l\right)}\left(m\right)y_{i}^{\left(l-1\right)}\left(m\right) \end{array}$$

where *τ* is the momentum constant, *η* is the learning rate.

  *m* ← *m* + 1.

 *end while*

### Adjustment of the output label values

The SAHRBF-BP algorithm can handle binary class problems and multi-class problems. For multi-class classification problems, suppose that the observation dataset is given as ${\left\{x_{n},y_{n}\right\}}_{n=1}^{N}$, where **x**_**n**_ ∈ *R*^*t*^ is an *t*–dimentional observation features and **y**_**n**_ ∈ *R*^*h*^ is its coded class label. Here, *h* is the total number of classes, which is equal to the number of output hidden neurons. If the observation data **x**_**n**_ is assigned to the class label *c*, then the *cth* element of **y**_**n**_ = [*y*_1_, …, *y*_*c*_, … *y*_*h*_]^*T*^ is 1 and other elements are -1, which can be denoted as follows:

$$\begin{array}{c} y_{j}=\left\{ \begin{array}{cc} 1 & \mathrm{if}\;j=c\\ -1 & \mathrm{otherwise} \end{array}\right.\;j=1,2,...,h \end{array}$$ (19)

The output tags of SAHRBF-BP are $\hat{y}={\left[{\hat{y}}_{1},...,{\hat{y}}_{c},...{\hat{y}}_{h}\right]}^{T}$, where

$$\begin{array}{c} {\hat{y}}_{j}=\text{sgn}\left(o_{j}\right),\;\;j=1,2,...h \end{array}$$ (20)

According to the coding rules, only one output tag value is 1, and the other value is -1. If this condition is not met, the output tag is saturated and must be adjusted. Therefore, we set an effective way to correct the saturation problem in the learning process, which can be denoted as the pseudo code in Algorithm 2.

Algorithm 2 The method of adjusting the output saturation problem

 Given observation dataset ${\left\{x_{n},y_{n}\right\}}_{n=1}^{N}$, for every input vector **x**_**n**_,

  *while* *j* ≤ *h*

   *if* the number of ${\hat{y}}_{j}==-1$ is equal to *h*

    Set max(*o*_*j*_) = 1 and and hold other output values fixed.

   *end if*

   *if* the number of ${\hat{y}}_{j}==1$ is more than 1

    Set max(*o*_*j*_) = 1 and the other output values are -1.

   *end if*

  *end while*

## Results

In this section, we evaluate the performance of SAHRBF-BP using two artificial data sets and 108 benchmark data sets, where Double moon data set are taken from [39], 101 benchmark data sets are taken from the UCI machine learning repository [40]. In addition, seven benchmark data sets including cod_rna, DNA, fourclass, ijcnn1, splice, svmguide1 and svmguide3 are taken from [41]. Tables 1–3 provide descriptions of the benchmark data sets. The benchmark data sets are grouped into three categories: binary class, multi-class and large number of samples. All benchmark binary class and multi-class benchmark data sets are grouped into low dimensional and high dimensional sets. For all benchmark data sets, the inputs to each algorithm are scaled appropriately to fall between -1 and +1. In each data set, the training set, validation set and testing set are independent. For balanced data sets, the number of training samples in each class is identical, which is also applicable to the validation set and testing set. For imbalance data sets, when the number of training samples is given, the number of each class of training samples is determined according to the proportion of each class in the whole data set. This method is also applicable to the validation set and testing set.

**Table 1:** Descriptions of binary class data sets.

Index	Data sets	# Classes	# Features	# Training	# Validation	# Testing
B01	Australian	2	14	345	172	173
B02	Banknote	2	4	686	343	343
B03	Blood	2	4	374	187	187
B04	Breast cancer	2	9	138	70	69
B05	Breast(original)	2	9	350	174	175
B06	Cleve	2	13	148	74	74
B07	Diabetes	2	8	576	100	92
B08	Fertility	2	9	50	30	20
B09	Fourclass	2	2	432	116	115
B10	Haberman	2	3	153	76	77
B11	Heart disease	2	13	151	76	76
B12	Liver	2	6	172	87	86
B13	Mammographic	2	5	480	241	240
B14	Monk1	2	6	124	100	332
B15	Monk2	2	6	169	100	332
B16	Monk3	2	6	122	100	332
B17	Planning	2	12	91	46	45
B18	Svmguide1	2	4	3089	1500	2500
B19	Vertebral	2	6	105	52	53
B20	Wholesale	2	7	220	110	110
B21	Wilt	2	6	3000	1339	500
B22	Breast(diagnostic)	2	30	284	143	142
B23	Breast(prognostic)	2	33	100	49	49
B24	Chronic	2	24	200	100	100
B25	Climate	2	18	270	135	135
B26	Congressional	2	16	210	100	125
B27	First order	2	51	3059	800	730
B28	Hill	2	100	606	303	303
B29	Hill(with noise)	2	100	606	303	303
B30	German	2	24	500	250	250
B31	Ionosphere	2	34	175	88	88
B32	LSVT	2	310	63	32	31
B33	Mushrooms	2	21	3000	1500	3624
B34	Musk1	2	167	238	119	119
B35	Musk2	2	167	3200	1600	1598
B36	Parkinsons	2	20	97	49	49
B37	QSAR	2	41	527	264	264
B38	Retinopathy	2	19	575	288	288
B39	Secom	2	591	783	392	392
B40	Seismic bumps	2	18	1292	646	646
B41	Sonar	2	60	104	52	52
B42	Spambase	2	57	2300	1151	1150
B43	Spect heart	2	22	80	47	140
B44	Splice	2	60	1000	500	1675
B45	Svmguide3	2	22	1000	243	41
B46	Vote	2	16	217	109	109

**Table 2:** Descriptions of multi-class data sets.

Index	Data sets	# Classes	# Features	# Training	# Validation	# Testing
M01	Balance	3	4	300	150	175
M02	Breast tissue	6	9	53	27	26
M03	Car	4	6	864	432	432
M04	Cardiotocography	10	9	1063	532	531
M05	Contraceptive	3	9	736	369	368
M06	Ecoli	8	7	168	84	84
M07	Glass	6	9	109	53	52
M08	Hayes-Roth	3	5	100	32	28
M09	Iris	3	4	75	36	39
M10	Knowledge	4	5	130	128	145
M11	Page	5	10	2737	1368	1368
M12	Seeds	3	7	105	53	52
M13	Teaching	3	5	75	38	38
M14	Vowel	11	10	264	132	132
M15	Wine	3	13	90	43	45
M16	Wine quality(red)	6	12	800	400	399
M17	Wine quality(white)	7	12	2450	1000	3448
M18	Yeast	8	10	742	371	371
M19	Air	3	64	178	81	100
M20	Dermatology	6	34	183	92	91
M21	DNA	3	180	2000	593	593
M22	Firm	4	16	5400	2700	2700
M23	Forest	4	27	198	100	225
M24	Gas(2012)	6	128	2919	1000	1000
M25	Image segmentation	7	19	210	420	1680
M26	Landsat	6	36	4435	1000	1000
M27	Libras	15	90	300	30	30
M28	Optical digits	10	64	3823	900	897
M29	Semeion	10	256	796	400	397
M30	Steel	7	20	971	385	385
M31	Turkiye	3	32	2910	1455	1455
M32	Vehicle silhouettes	4	18	424	211	211
M33	Waveform1	3	21	2500	1250	1250
M34	Waveform2	3	40	2500	1250	1250
M35	Zoo	16	101	51	25	25

**Table 3:** Descriptions of large number of samples data sets.

Index	Data sets	# Classes	# Features	# Training	# Validation	# Testing
L01	A1a(adult)	2	14	1605	957	30000
L02	A6a(adult)	2	14	11221	5000	16342
L03	Action1(normal)	10	8	20000	10000	68886
L04	Action2(normal)	10	8	15000	10000	74930
L05	Action3(normal)	10	8	15000	10000	74872
L06	Action4(normal)	10	8	15000	10000	74069
L07	Action1(aggressive)	10	8	15000	10000	73172
L08	Action2(aggressive)	10	8	15000	10000	74690
L09	Action3(aggressive)	10	8	10000	5000	89543
L10	Action4(aggressive)	10	8	10000	5000	82764
L11	Action1(abnormal detection)	2	8	15000	5000	177058
L12	Action2(abnormal detection)	2	8	20000	10000	169620
L13	Action3(abnormal detection)	2	8	20000	15000	179415
L14	Action4(abnormal detection)	2	8	20000	10000	166833
L15	Cod_rna	2	8	15220	10000	20000
L16	Credit	2	23	10000	5000	15000
L17	Eye	2	14	7490	3745	3745
L18	Gas(2013)	6	128	6910	3500	3500
L19	Ijcnn1	2	13	15000	5000	15000
L20	Letter	26	16	10000	5000	5000
L21	Occupancy	2	5	8143	2665	9752
L22	Pendigits	10	16	7495	2000	1498
L23	Record	2	7	20000	10000	94913
L24	Sensorless	11	48	11000	7700	39809
L25	Skin	2	3	25000	10957	210000
L26	Shuttle	5	9	33500	10000	14500
L27	Telescope	2	10	9510	5000	4510

The performance of SAHRBF-BP is compared with other well-known training and optimization SLFNs algorithms, such as SGBP, MRAN, SVM, ELM, and SaE-ELM on different data sets. To measure the unique features of SAHRBF-BP, other optimization algorithms such as KMRBF, GAP-RBF and KMRBF-BP are also compared to SAHRBF-BP on two artificial data sets, namely Double moon and Concentric circle. For SGBP, the momentum constant is set to *τ* = 0.1. For SVM, the RBF is used as the kernel function, the cost *C* is selected from the set [2^12^, 2^11^, …, 1] and kernel parameter is selected from the set [2^−3^, 2^−2^, …, 2^4^]. For GAP-RBF and MRAN, the common parameters are fixed to *ε*_max_ = 0.5, *ε*_min_ = 0.01, *k* = 0.8 and *γ* = 0.09. Other parameters for GAP-RBF are set to *e*_min_ = 0.01; for MRAN, the parameters are set to *e*_min_ = 0.5, $e_{\min}^{\prime }=0.3$, the sliding window *M* is selected from the set [30, 50, 100, 200, 400]. For SaE-ELM, the number of populations *NP* is selected from the set [20, 50, 100, 200, 500]. For SAHRBF-BP, the common parameters of the distance weighting factor, width covering factor, width constraint factor, potential value learning threshold are set to *T* = 1, *λ* = 1.5, *β* = 1.3, *δ* = 0.001, respectively. The heterogeneous sample repulsive force control factor *α* is selected from the set [2, 5, 10, 15, 20]. The initial width *σ* is selected from the set [0.4, 0.5, …, 1.6]. Note the number of hidden nodes in KMRBF, KMRBF-BP and SaE-ELM is selected manually. When gradually increasing the number of hidden nodes, the one with the lowest overall validation error is selected as the number of hidden nodes. For benchmark data sets with the number of training samples is less than 2000 and artificial data sets, simulations in each algorithm are performed 20 times and are conducted in the MATLAB 2013a environment on an Intel(R) Core(TM) i5 with a 3.2GHZ CPU and 4G of RAM. For other data sets, simulations in each algorithm are performed 3∼15 times and are conducted in the MATLAB 2013a environment on an Intel(R) Xeon(R) CPU E5-2687w @3.40GHZ(dual processor) and 128G of RAM. For each algorithm, the one with the lowest validation error is used to determine the parameters in the training models. The simulations for the SVM are carried out using the popular LIBSVM package in C [41].

### Performance measures

In this paper, the overall and average per-class classification accuracies are used to measure performance. Class-level performance is measured by the percentage classification (*η*_*i*_), which is defined as

$$\begin{array}{c} \eta_{i}=\frac{q_{ii}}{N_{i}^{T}} \end{array}$$ (21)

where *q*_*ii*_ is the number of correctly classified samples and $N_{i}^{T}$ is the number of samples for the class **y**_**i**_ in the training/testing data set. The overall classification accuracy(*η*_*o*_) and the average per-class classification accuracy(*η*_*a*_) are defined as

$$\begin{array}{c} \eta_{o}=100\times \frac{1}{N^{T}}\sum_{i=1}^{h}q_{ii} \end{array}$$ (22)

$$\begin{array}{c} \eta_{a}=100\times \frac{1}{h}\sum_{i=1}^{h}\eta_{i} \end{array}$$ (23)

where *h* is the number of classes, *N*^*T*^ is the number of training/testing samples. Thus, for balanced classification problems, the overall testing *η*_*o*_ is used to measure the performance of each algorithm. For imbalanced classification problems, the overall testing *η*_*o*_ and the average testing *η*_*a*_ are used to measure the performance of each algorithm.

### Performance comparison

#### Artificial binary class data set: The Double moon classification problem

**Fig 6:**
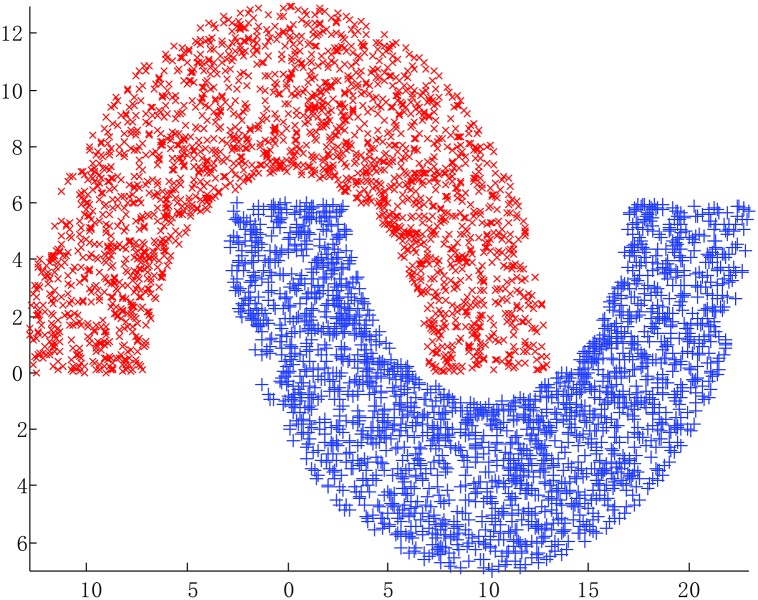
Double moon classification problem. (A) Double moon data set (B) Classifying result of SAHRBF-BP.

**Fig 7:**
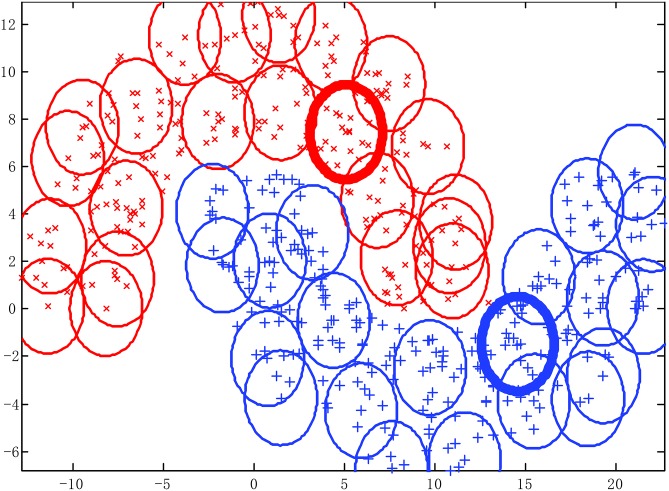
Using different width parameters to cover the training sample space for Double moon classification problem. (A) *σ* = 2 (B) *σ* = 3 (C) *σ* = 4.

The Double moon data set and the classifying results of SAHRBF-BP are shown in Fig 6(A) and 6(B), respectively. The classification results illustrate that SAHRBF-BP can provide a superior classification surface. Fig 7(A)–7(C) show when the initial width takes different values, the adaptive coverage of the training sample space can be completed effectively. Each cover generates a RBF hidden node and the number of RBF hidden nodes is increased incrementally, the bold lines represent the first coverage region in each pattern class, which can be seen as the densest region learned by the potential function clustering approach. With the change of the initial width, the number of RBF hidden nodes and node parameters are changed accordingly. Based on the potential function clustering to generate initial RBF hidden nodes, the form of heterogeneous sample repulsive force can further ensure that each initial RBF hidden node adjusted to a suitable position. In this way, the optimal coverage of the training sample space can be completed and the RBF centers and the width and number of RBF hidden nodes can be effectively estimated.

**Fig 8:**
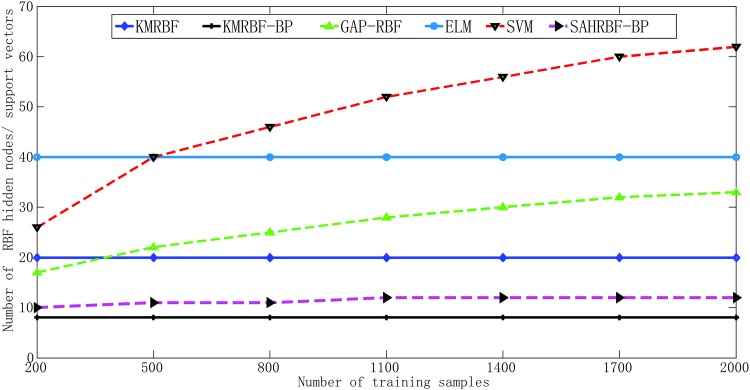
Performance comparisons between SAHRBF-BP and other algorithms on Double moon data set. (A) Number of training samples- Number of RBF hidden neurons/ support vectors (B) Number of training samples- Overall classifying accuracy.

In Fig 8, when the number of training samples has changed, KMRBF-BP needs less number of RBF hidden neurons than KM-RBF and can get a higher classifying accuracy. This results demonstrate the hybrid RBF-BP network structure is effective, which can improve the classifying accuracy and reduce the dependence on the original sample space mapping. Note the number of KM-RBF and KMRBF-BP is selected manually, when changing the number of hidden neurons several times, the one with the highest overall validation accuracy is selected as the suitable number of hidden neurons. The classifying accuracy of SAHRBF-BP is comparable with KMRBF-BP, however, the number of RBF hidden nodes in SAHRBF-BP is generated adaptively. The classifying accuracy of SAHRBF-BP outperforms SGBP apparently, which further shows the effectiveness of SAHRBF-BP, the reason is that the structure-adaptive RBF network can improve the separability of sample spaces. Compared to GAP-RBF, SAHRBF-BP can better adapt to the change of sample space. The classifying accuracy of SAHRBF-BP outperforms GAP-RBF and needs less RBF hidden nodes. The classifying accuracy of SAHRBF-BP is comparable with SVM, however, the number of RBF hidden nodes in SAHRBF-BP is less than SVM apparently. Thus, SAHRBF-BP can adapt training sample space well, which can get a high classifying accuracy, as well as a compact network size for the RBF hidden layer.

#### Artificial binary class data set: The Concentric circle classification problem

**Fig 9:**
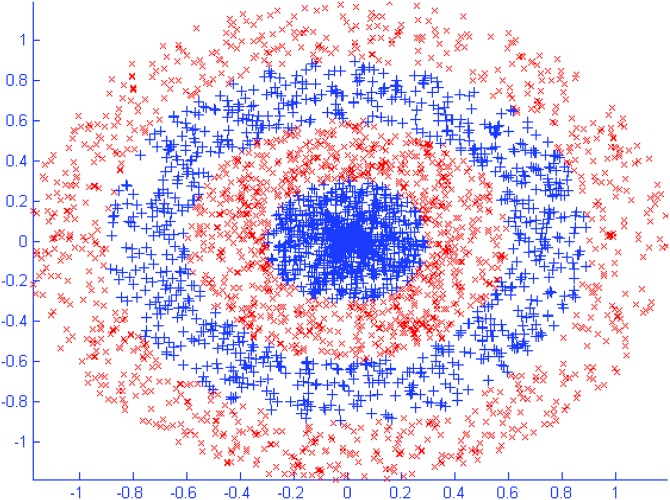
Concentric circle classification problem. (A) Concentric circle data set (B) Classifying result of SAHRBF-BP.

The Concentric circle data set and the classifying results of SAHRBF-BP are shown in Fig 9(A) and 9(B), respectively. Compared to the Double moon classification problem, the Concentric circle classification problem is more complex and can thus be used to measure the unique features of SAHRBF-BP. The classification results illustrate SAHRBF-BP can still provide a superior classification surface for Concentric circle classification problem.

**Fig 10:**
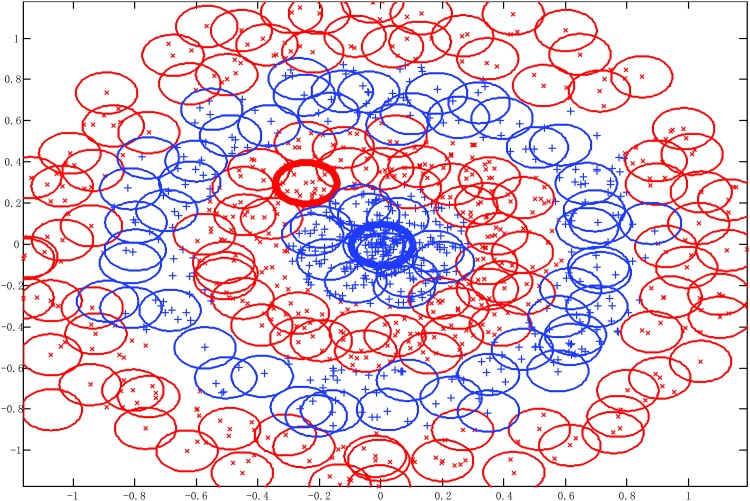
Using different width parameters to cover the training sample space for Concentric circle classification problem. (A) *σ* = 0.1 (B) *σ* = 0.2 (C) *σ* = 0.3.

Fig 10(A)–10(C) show when the initial width takes different values, the adaptive coverage of the training sample space can be completed effectively. Each cover generates a RBF hidden node and the number of RBF hidden nodes is increased incrementally, the bold lines represent the first coverage region in each pattern class. With the change of the initial width, the number of RBF hidden nodes and node parameters change accordingly. For each generated initial RBF hidden node, the form of heterogeneous sample repulsive force can further ensure each initial RBF hidden node adjusted to a suitable position. Thus, the optimal coverage of the training sample space can be completed and the RBF centers and the width and number of RBF hidden nodes can be effectively estimated.

**Fig 11:**
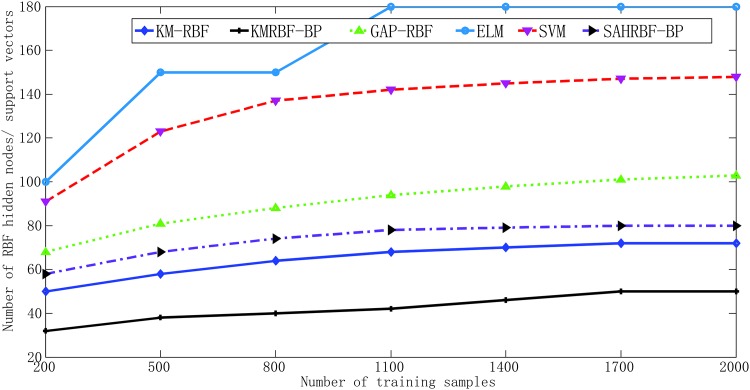
Performance comparisons between SAHRBF-BP and other algorithms on Concentric circle data set. (A) Number of training samples- Number of RBF hidden neurons/ support vectors (B) Number of training samples- Overall classifying accuracy.

Fig 11(A) and 11(B) demonstrate when the number of training samples changes, KMRBF-BP needs less number of RBF hidden neurons than KM-RBF and can get a higher classifying accuracy. Thus the hybrid RBF-BP network architecture improves the classifying accuracy and reduces the dependence on the original sample space mapping. Note in KM-RBF and KMRBF-BP, when the number of training samples changes, the number of RBF hidden neurons has to be adjusted manually, otherwise it will lead to a poor classification accuracy. Compared with KM-RBF and KMRBF-BP, SAHRBF-BP can adapt the training sample space well, when the number of training samples changes, the number of RBF hidden neurons in SAHRBF-BP changes accordingly, and can get a higher classifying accuracy. Compared to GAP-RBF, SAHRBF-BP can better adapt to the change of sample space. The classifying accuracy of SAHRBF-BP outperforms GAP-RBF and ELM apparently. When the number of training samples is more than 500, the classifying accuracy of SAHRBF-BP outperforms SVM. In this way, the effectiveness of SAHRBF-BP is further verified.

For the Concentric circle classification problem, SAHRBF-BP can get a higher classifying accuracy than other training SLFNs algorithms, however, there are still a certain number of incorrect predictions in SAHRBF-BP. In Fig 9(B), we can see the samples of incorrect predictions generally appear in the boundary region. A main reason is that, due to the complexity of the sample set, it is often difficult to achieve an ideal coverage of the sample space. As shown in Fig 10(A)–10(C), if the current class of RBF hidden nodes cover heterogeneous samples, it may lead to a reduction in classification performance. From this point of view, to get a higher classification performance, it is necessary to optimize each generated RBF hidden node. In SAHRBF-BP, the combination of potential function clustering and heterogeneous samples repulsive force can adaptively determine the number of RBF hidden nodes and optimize node parameters, which ensures each generated RBF hidden node covers the samples of the current class as much as possible, while covering heterogeneous samples as little as possible.

**Fig 12:**
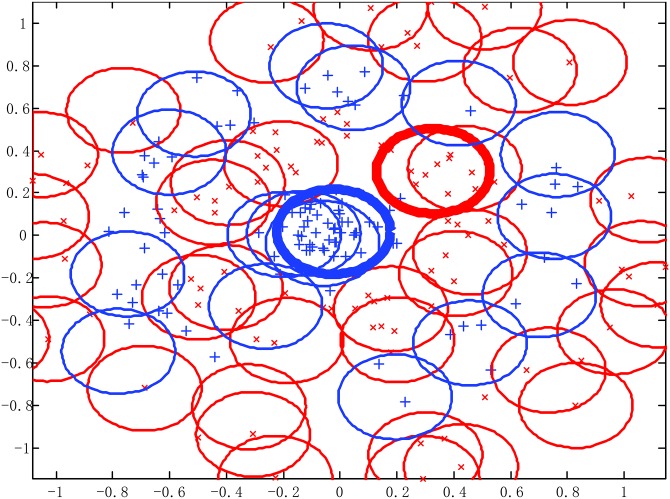
The learning effect of training and testing sample space when the number of training samples is 200. (A) Covering effect of training sample space (B) Classifying result of SAHRBF-BP.

In Fig 11(B), when the number of training samples is reduced, it will lead to a reduction in classification performance. Especially when the number of training samples is 200, the overall classifying accuracy of SAHRBF-BP is a little lower than SVM. Fig 12 further shows inadequate training samples lead to the reduction of the classification accuracy. For complex data sets, when the number of training samples is reduced, the randomness of training samples in the sample space is enhanced, which can not effectively reflect the actual distribution of the entire data set, and may lead to some extent of failure by the methods of potential function clustering and heterogeneous samples repulsive force.

#### Benchmark binary class classification problems

**Fig 13:**
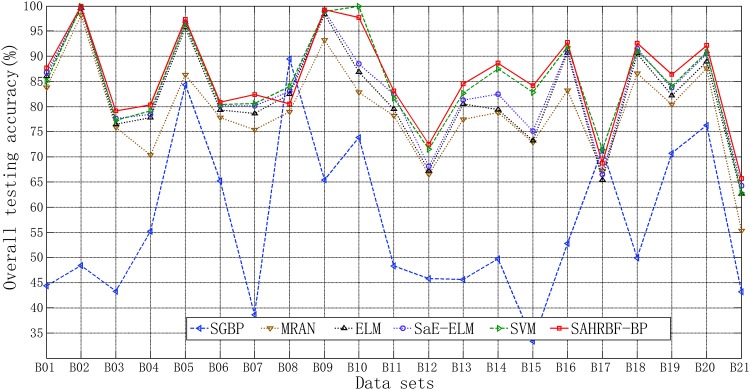
Overall testing accuracy comparisons between SAHRBF-BP and other algorithms on benchmark binary class data sets. (A) Binary class low dimensional data sets (B) Binary class high dimensional data sets.

In this section, 21 benchmark binary class low dimensional data sets and 25 benchmark binary class high dimensional data sets are used to evaluate the performance of SAHRBF-BP. Fig 13 shows the overall testing accuracy comparisons between SAHRBF-BP and other learning algorithms. For binary class low dimensional data sets, the overall testing accuracy of SAHRBF-BP is higher than other learning algorithms on most data sets, except for Fertility(B08), Haberman(B10) and Planning(B17) data sets. For binary class high dimensional data sets, the overall testing accuracy of SAHRBF-BP is higher than other learning algorithms on Breast(diagnostic)(B22), Chronic(B24), Climate(B25), Congressional(B26), First order(B27), German(B30), Ionosphere(B31), Retinopathy(B38), Spambase(B42) and Vote(B46) data sets. The overall testing accuracy of SAHRBF-BP is comparable with SVM on Mushrooms(B33), Musk2(B35), Seismic bumps(B40), Splice(B44) and Svmguide3(B45) data sets, however, the overall testing accuracy of SAHRBF-BP is lower than SVM on Musk1(B34), Parkinsons(B36), QSAR(B37), Secom(B39) and Sonar(B41) data sets, lower than ELM and SaE-ELM on Hill(with noise)(B29) data set, lower than SVM, ELM and SaE-ELM on Hill(B28) data set, and lower than SGBP and SVM on Breast(prognostic)(B23) and Spect heart(B43) data sets.

Tables 4–6 give performance comparisons between SAHRBF-BP and other learning algorithms, where a few cases of success and failures are given a more detail description. In Tables 4 and 5, the overall and average testing accuracies of SAHRBF-BP are clearly higher than SGBP. For Blood data set, the overall testing accuracy of SAHRBF-BP outperforms SVM by approximately 1.8%, and SaE-ELM, ELM, MRAN by approximately 1.5%-3.3%. The average testing accuracy of SAHRBF-BP outperforms SVM by approximately 2.2%, and SaE-ELM, ELM, MRAN by approximately 1.4%-4.5%.

**Table 4:** A few cases of success in SAHRBF-BP compared with other learning algorithms on benchmark binary class data sets.

Index	Data sets	Methods	Testing *η*_*o*_	Testing *η*_*a*_	*N*_*H*_ nodes
B03	Blood	SGBP	43.29	48.74	7
		MRAN	75.83	73.21	177
		ELM	76.48	75.32	80
		SaE-ELM	77.64	76.28	40
		SVM	77.27	75.53	265^a^
		SAHRBF-BP	79.12	77.69	113&7^b^
B07	Diabetes	SGBP	38.62	52.58	7
		MRAN	75.39	74.73	41
		ELM	78.64	78.36	50
		SaE-ELM	80.13	79.72	22
		SVM	80.63	77.41	301^a^
		SAHRBF-BP	82.37	82.65	19&4^b^
B11	Heart disease	SGBP	48.37	48.76	8
		MRAN	78.16	77.94	29
		ELM	79.56	78.62	30
		SaE-ELM	82.52	82.42	20
		SVM	81.70	85.85	42^a^
		SAHRBF-BP	83.13	83.64	12&4^b^
B13	Mammographic	SGBP	45.65	53.81	8
		MRAN	77.49	75.28	86
		ELM	80.54	79.15	50
		SaE-ELM	81.32	80.42	30
		SVM	82.64	82.28	196^a^
		SAHRBF-BP	84.52	83.74	26&6^b^
B14	Monk1	SGBP	49.77	48.31	8
		MRAN	78.51	77.42	32
		ELM	79.40	77.84	30
		SaE-ELM	82.52	81.28	20
		SVM	87.50	86.21	56^a^
		SAHRBF-BP	88.64	87.83	23&8^b^
B15	Monk2	SGBP	33.35	37.73	7
		MRAN	72.86	70.66	58
		ELM	73.12	72.58	50
		SaE-ELM	75.15	74.57	30
		SVM	82.87	83.14	72^a^
		SAHRBF-BP	84.21	83.65	34&6^b^
B18	Svmguide1	SGBP	49.98	62.63	20
		MRAN	86.56	85.16	621
		ELM	90.52	90.36	200
		SaE-ELM	91.49	90.82	160
		SVM	91.15	90.74	2096^a^
		SAHRBF-BP	92.62	92.28	442&9^b^
B19	Vertebral	SGBP	70.74	76.42	6
		MRAN	80.43	78.51	34
		ELM	82.25	81.46	40
		SaE-ELM	83.72	83.19	30
		SVM	84.12	83.80	71^a^
		SAHRBF-BP	86.41	85.23	45&6^b^

^a^Support vectors.

^b^RBF&BP hidden nodes.

**Table 5:** A few cases of success in SAHRBF-BP compared with other learning algorithms on benchmark binary class data sets.

Index	Data sets	Methods	Testing *η*_*o*_	Testing *η*_*a*_	*N*_*H*_ nodes
B20	Wholesale	SGBP	76.27	74.58	8
		MRAN	87.55	85.21	41
		ELM	88.92	87.31	60
		SaE-ELM	90.41	89.45	30
		SVM	90.84	90.42	55^a^
		SAHRBF-BP	92.17	91.54	36&5^b^
B25	Climate	SGBP	91.48	90.62	7
		MRAN	88.26	86.68	27
		ELM	91.85	91.53	50
		SaE-ELM	92.51	91.94	30
		SVM	92.32	92.64	49^a^
		SAHRBF-BP	93.47	92.41	13&5^b^
B26	Congressional	SGBP	48.53	52.79	7
		MRAN	92.59	90.31	47
		ELM	95.26	94.72	50
		SaE-ELM	95.81	95.27	40
		SVM	94.75	94.16	97^a^
		SAHRBF-BP	96.82	96.58	26&4^b^
B30	German	SGBP	22.92	39.16	8
		MRAN	68.45	66.29	137
		ELM	75.54	70.50	120
		SaE-ELM	77.51	72.63	80
		SVM	77.38	76.39	234^a^
		SAHRBF-BP	82.62	81.54	262 &9^b^
B31	Ionosphere	SGBP	73.30	56.86	8
		MRAN	84.34	82.29	86
		ELM	89.77	85.93	100
		SaE-ELM	90.75	88.14	60
		SVM	91.26	91.82	50^a^
		SAHRBF-BP	93.52	94.04	54&6^b^
B38	Retinopathy	SGBP	41.49	53.82	7
		MRAN	70.54	72.28	152
		ELM	73.16	72.75	100
		SaE-ELM	74.62	74.56	50
		SVM	74.31	76.87	339^a^
		SAHRBF-BP	77.25	78.51	107&7^b^
B42	Spambase	SGBP	57.02	60.32	9
		MRAN	85.28	86.56	421
		ELM	90.32	91.54	100
		SaE-ELM	91.82	91.60	70
		SVM	91.67	91.21	862^a^
		SAHRBF-BP	93.21	92.79	284&9^b^
B46	Vote	SGBP	73.26	75.82	6
		MRAN	90.15	89.21	26
		ELM	93.72	93.27	40
		SaE-ELM	94.36	94.13	20
		SVM	93.58	94.28	31^a^
		SAHRBF-BP	95.12	95.34	15&4^b^

^a^Support vectors.

^b^RBF&BP hidden nodes.

**Table 6:** A few cases of failures in SAHRBF-BP compared with other learning algorithms on benchmark binary class data sets.

Index	Data sets	Methods	Testing *η*_*o*_	Testing *η*_*a*_	*N*_*H*_ nodes
B08	Fertility	SGBP	89.50	86.52	7
		MRAN	79.00	77.36	25
		ELM	82.50	81.28	40
		SaE-ELM	83.00	81.85	20
		SVM	84.00	82.94	18^a^
		SAHRBF-BP	80.50	78.42	11&4^b^
B17	Planning	SGBP	71.39	70.21	6
		MRAN	67.24	65.86	24
		ELM	65.47	66.23	30
		SaE-ELM	66.52	66.58	20
		SVM	71.43	70.52	69^a^
		SAHRBF-BP	68.76	67.34	24&5^b^
B28	Hill	SGBP	51.65	50.52	10
		MRAN	52.38	51.61	226
		ELM	55.91	54.32	120
		SaE-ELM	56.53	55.83	80
		SVM	55.94	54.76	551^a^
		SAHRBF-BP	53.65	52.67	243&8^b^
B32	LSVT	SGBP	33.33	46.51	7
		MRAN	71.58	69.42	38
		ELM	73.14	72.41	50
		SaE-ELM	74.55	72.87	20
		SVM	85.79	86.61	53^a^
		SAHRBF-BP	72.52	71.86	12&4^b^
B34	Musk1	SGBP	43.28	43.79	9
		MRAN	75.57	74.87	93
		ELM	74.79	74.52	60
		SaE-ELM	75.81	75.25	30
		SVM	88.03	88.26	147^a^
		SAHRBF-BP	79.26	78.82	117&7^b^
B37	QSAR	SGBP	46.71	44.53	8
		MRAN	82.27	80.72	107
		ELM	84.62	85.28	80
		SaE-ELM	85.64	85.80	60
		SVM	87.16	86.52	192^a^
		SAHRBF-BP	85.71	84.85	43&5^b^
B41	Sonar	SGBP	46.09	47.17	8
		MRAN	67.26	65.29	74
		ELM	70.37	70.06	50
		SaE-ELM	70.24	69.62	30
		SVM	80.85	84.97	46^a^
		SAHRBF-BP	77.74	76.59	49&5^b^
B43	Spect heart	SGBP	91.98	90.21	8
		MRAN	66.72	65.63	32
		ELM	66.24	65.56	50
		SaE-ELM	68.03	67.83	20
		SVM	70.05	72.52	78^a^
		SAHRBF-BP	67.58	65.84	14&4^b^

^a^Support vectors.

^b^RBF&BP hidden nodes.

For Diabetes data set, the overall testing accuracy of SAHRBF-BP outperforms SVM by approximately 1.7%, and SaE-ELM, ELM, MRAN by approximately 2.2%-7%. The average testing accuracy of SAHRBF-BP outperforms SVM by approximately 5.2%, and SaE-ELM, ELM, MRAN by approximately 2.9%-7.9%.

For Heart disease data set, the overall and average testing accuracies of SAHRBF-BP outperforms SaE-ELM, ELM, MRAN by approximately 0.6%-5.7%. The average testing accuracy of SAHRBF-BP is approximately 2.2% lower than that of SVM, however, the overall testing accuracy is higher than that of SVM by approximately 1.4%.

For Mammographic data set, the overall testing accuracy of SAHRBF-BP outperforms SVM by approximately 1.9%, and SaE-ELM, ELM, MRAN by approximately 3.2%-7%. The average testing accuracy of SAHRBF-BP outperforms SVM by approximately 1.5%, and SaE-ELM, ELM, MRAN by approximately 3.3%-8.5%.

For Monk1 data set, the overall testing accuracy of SAHRBF-BP outperforms SVM by approximately 1.1%, and SaE-ELM, ELM, MRAN by approximately 6.1%-10.1%. The average testing accuracy of SAHRBF-BP outperforms SVM by approximately 1.6%, and SaE-ELM, ELM, MRAN by approximately 6.5%-10.4%.

For Monk2 data set, the overall testing accuracy of SAHRBF-BP outperforms SVM by approximately 1.3%, and SaE-ELM, ELM, MRAN by approximately 9%-11.3%. The average testing accuracy of SAHRBF-BP outperforms SVM by approximately 0.5%, and SaE-ELM, ELM, MRAN by approximately 9.1%-13%.

For Svmguide1 data set, the overall testing accuracy of SAHRBF-BP outperforms SVM by approximately 1.6%, and SaE-ELM, ELM, MRAN by approximately 2.1%-6%. The average testing accuracy of SAHRBF-BP outperforms SVM by approximately 1.5%, and SaE-ELM, ELM, MRAN by approximately 1.4%-7.1%.

For Vertebral data set, the overall testing accuracy of SAHRBF-BP outperforms SVM by approximately 2.3%, and SaE-ELM, ELM, MRAN by approximately 2.7%-6%. The average testing accuracy of SAHRBF-BP outperforms SVM by approximately 1.4%, and SaE-ELM, ELM, MRAN by approximately 2%-6.7%.

For Wholesale data set, the overall testing accuracy of SAHRBF-BP outperforms SVM by approximately 1.3%, and SaE-ELM, ELM, MRAN by approximately 1.8%-4.6%. The average testing accuracy of SAHRBF-BP outperforms SVM by approximately 1.1%, and SaE-ELM, ELM, MRAN by approximately 2.1%-6.3%.

For Climate data set, the overall testing accuracy of SAHRBF-BP outperforms SVM by approximately 1.1%, and SaE-ELM, ELM, MRAN by approximately 1%-5.2%. The average testing accuracy of SAHRBF-BP is comparable with SVM, and outperforms SaE-ELM, ELM, MRAN by approximately 0.5%-5.7%.

For Congressional data set, the overall testing accuracy of SAHRBF-BP outperforms SVM by approximately 2.1%, and SaE-ELM, ELM, MRAN by approximately 1%-4.2%. The average testing accuracy of SAHRBF-BP outperforms SVM by approximately 2.4%, and SaE-ELM, ELM, MRAN by approximately 1.3%-6.3%.

For German data set, the overall testing accuracy of SAHRBF-BP outperforms SVM by approximately 5.2%, and SaE-ELM, ELM, MRAN by approximately 5.1%-14.2%. The average testing accuracy of SAHRBF-BP outperforms SVM by approximately 5.1%, and SaE-ELM, ELM, MRAN by approximately 8.9%-15.2%.

For Ionosphere data set, the overall testing accuracy of SAHRBF-BP outperforms SVM by approximately 2.2%, and SaE-ELM, ELM, MRAN by approximately 2.8%-9.2%. The average testing accuracy of SAHRBF-BP outperforms SVM by approximately 2.2%, and SaE-ELM, ELM, MRAN by approximately 5.9%-11.7%.

For Retinopathy data set, the overall testing accuracy of SAHRBF-BP outperforms SVM by approximately 2.9%, and SaE-ELM, ELM, MRAN by approximately 2.6%-6.7%. The average testing accuracy of SAHRBF-BP outperforms SVM by approximately 1.6%, and SaE-ELM, ELM, MRAN by approximately 4%-6.2%.

For Spambase data set, the overall testing accuracy of SAHRBF-BP outperforms SVM by approximately 1.5%, and SaE-ELM, ELM, MRAN by approximately 1.4%-8%. The average testing accuracy of SAHRBF-BP outperforms SVM by approximately 1.6%, and SaE-ELM, ELM, MRAN by approximately 1.2%-6.2%.

For Vote data set, the overall testing accuracy of SAHRBF-BP outperforms SVM by approximately 1.5%, and SaE-ELM, ELM, MRAN by approximately 0.8%-5%. The average testing accuracy of SAHRBF-BP outperforms SVM by approximately 1%, and SaE-ELM, ELM, MRAN by approximately 1.2%-6.1%.

In Table 6, for Fertility data set, the overall testing accuracy of SAHRBF-BP is lower than SGBP about 9%, lower than SVM about about 3.5%, SaE-ELM about 2.5%, and ELM about 2%. The average testing accuracy of SAHRBF-BP is lower than SGBP about 8.1%, and SVM, SaE-ELM, ELM about 4.5%, 3.4%, 2.8%, respectively. For Planning data set, the overall and average testing accuracies are lower than SGBP and SVM about 2.6%-3.2%. For Hill data set, the overall testing accuracy is lower than ELM, SVM and SaE-ELM about 2.3%-2.9%, and the average testing accuracy of SAHRBF-BP is lower than ELM, SVM, SaE-ELM about 1.7%-3.1%. For LSVT data set, the overall testing accuracy is lower than ELM about 0.6%, SaE-ELM about 2%, and SVM about 13.3%. The average testing accuracy of SAHRBF-BP is lower than ELM, SaE-ELM and SVM about 0.7%, 1%, 14.8%, respectively. For Musk1 data set, the overall and average testing accuracies are lower than SVM about 8.8% and 9.4%, respectively. For QSAR data set, the overall and average testing accuracies are lower than SVM about 1.4% and 1.7%, respectively. For Sonar data set, the overall testing accuracy of SAHRBF-BP is lower than SVM about 3.1%, and the average testing accuracy of SAHRBF-BP is lower than SVM about 8.4%. For Spect heart data set, the overall and average testing accuracies are lower than SGBP clearly. The overall testing accuracy of SAHRBF-BP is lower than SaE-ELM, SVM about 0.5%, 2.5%, respectively. The average testing accuracy of SAHRBF-BP is lower than SaE-ELM and SVM about 2%, 6.7%, respectively.

From Fig 13 and Tables 4–6 we can see that for most binary class low dimensional data sets, the classification accuracy of SAHRBF-BP is higher than other learning algorithms. However, for binary class high dimensional data sets, the classification accuracy of SAHRBF-BP is decreased clearly on a number of data sets, such as LSVT(B32), Musk1(B34), QSAR(B37), Secom(B39), Sonar(B41) data sets. The main reasons are that, with the increase of dimension, spatial distribution of the samples is relatively sparse, especially for small number of training samples data sets, the randomness of training samples in the sample space is greatly enhanced, which can not effectively reflect the actual distribution of entire data sets, and leads to a certain degree of failure by the methods of potential function clustering and heterogeneous samples repulsive force. Thus, the classification performance of SAHRBF-BP will be reduced to varying degrees.

#### Benchmark multi-class classification problems

**Fig 14:**
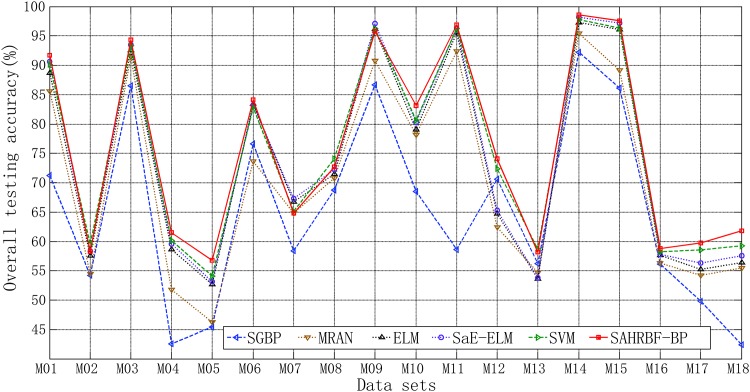
Overall testing accuracy comparisons between SAHRBF-BP and other algorithms on benchmark multi-class data sets. (A) Multi-class low dimensional data sets (B) Multi-class high dimensional data sets.

In this section, 18 multi-class low dimensional data sets and 17 multi-class high dimensional data sets are used to evaluate the performance of SAHRBF-BP. Fig 14 shows the overall testing accuracy comparisons between SAHRBF-BP and other learning algorithms. For multi-class low dimensional data sets, the overall testing accuracy of SAHRBF-BP is comparable with SVM on Teaching(M13) data set, and is lower than SVM on Breast tissue(M02), Hayes-Roth(M08) data sets, lower than SaE-ELM, ELM on Glass(M07) data set, lower than SaE-ELM, SVM, ELM on Iris(M09) data set, however, the overall testing accuracy of SAHRBF-BP is higher than other learning algorithms on the rest 13 data sets.

For multi-class high dimensional data sets, the overall testing accuracy of SAHRBF-BP is higher than other learning algorithms on Firm(M22), Image segmentation(M25), Landsat(M26), Steel(M30), Turkiye(M31), Vehicle silhouettes(M32), Waveform1(M33) and Waveform2(M34) data sets. The overall testing accuracy of SAHRBF-BP is comparable with SVM on Gas(2012)(M24) data set, and SVM, ELM, SaE-ELM on Optical digits(M28) and Semeion(M29) data sets, however, the overall testing accuracy of SAHRBF-BP is lower than SVM on Air(M19), DNA(M21), Forest(M23) and Libras(M27) data sets, and ELM, SaE-ELM, SVM on Dermatology(M20) and Zoo(M35) data sets.

Tables 7–9 give performance comparisons between SAHRBF-BP and other learning algorithms on partial multi-class data sets, where the overall and average testing accuracies of SAHRBF-BP are clearly higher than SGBP. In Tables 7 and 8, for Balance data set, the overall testing accuracy of SAHRBF-BP outperforms SVM by approximately 1.5%, and SaE-ELM, ELM, MRAN by approximately 1%-6%. The average testing accuracy of SAHRBF-BP outperforms SVM by approximately 1.3%, and SaE-ELM, ELM, MRAN by approximately 1.5%-7.5%.

**Table 7:** A few cases of success in SAHRBF-BP compared with other learning algorithms on benchmark multi-class data sets.

Index	Data sets	Methods	Testing *η*_*o*_	Testing *η*_*a*_	*N*_*H*_ nodes
M01	Balance	SGBP	71.23	68.51	7
		MRAN	85.61	83.29	47
		ELM	88.72	86.64	50
		SaE-ELM	90.64	89.33	40
		SVM	90.13	89.52	65^a^
		SAHRBF-BP	91.67	90.85	29&4^b^
M04	Cardiotocography	SGBP	42.57	46.73	11
		MRAN	51.74	52.84	387
		ELM	58.66	56.51	300
		SaE-ELM	59.57	58.52	200
		SVM	60.21	61.75	1378^a^
		SAHRBF-BP	61.52	62.58	281&9^b^
M10	Knowledge	SGBP	68.52	65.40	7
		MRAN	78.25	76.13	65
		ELM	79.07	78.61	50
		SaE-ELM	80.24	79.25	30
		SVM	80.65	81.23	184^a^
		SAHRBF-BP	83.16	82.24	56&5^b^
M12	Seeds	SGBP	70.58	–	7
		MRAN	62.42	–	26
		ELM	64.76	–	30
		SaE-ELM	65.29	–	20
		SVM	72.47	–	47^a^
		SAHRBF-BP	74.12	–	13&4^b^
M15	Wine	SGBP	86.13	–	8
		MRAN	89.16	–	46
		ELM	96.12	–	30
		SaE-ELM	97.18	–	22
		SVM	96.36	–	32^a^
		SAHRBF-BP	97.54	–	16&5^b^
M18	Yeast	SGBP	42.26	40.53	9
		MRAN	55.52	54.72	323
		ELM	56.41	55.65	200
		SaE-ELM	57.53	56.33	120
		SVM	59.27	58.34	852^a^
		SAHRBF-BP	61.84	60.82	216&8^b^
M25	Image segmentation	SGBP	90.16	–	8
		MRAN	85.49	–	81
		ELM	90.31	–	100
		SaE-ELM	91.17	–	50
		SVM	90.56	–	96^a^
		SAHRBF-BP	92.23	–	32&6^b^
M26	Landsat	SGBP	74.83	78.84	12
		MRAN	87.26	85.42	462
		ELM	90.34	90.13	600
		SaE-ELM	90.82	90.38	350
		SVM	91.21	90.87	1618^a^
		SAHRBF-BP	91.94	91.82	312&9^b^

^a^Support vectors.

^b^RBF&BP hidden nodes.

**Table 8:** A few cases of success in SAHRBF-BP compared with other learning algorithms on benchmark multi-class data sets.

Index	Data sets	Methods	Testing *η*_*o*_	Testing *η*_*a*_	*N*_*H*_ nodes
M30	Steel	SGBP	68.57	66.79	9
		MRAN	64.56	62.82	372
		ELM	72.38	71.53	200
		SaE-ELM	72.85	71.61	130
		SVM	71.51	70.87	263^a^
		SAHRBF-BP	73.72	72.86	274&9^b^
M31	Turkie	SGBP	45.84	42.12	14
		MRAN	53.25	52.87	534
		ELM	61.50	60.64	200
		SaE-ELM	62.27	61.89	100
		SVM	62.82	62.58	3237^a^
		SAHRBF-BP	64.26	63.80	415&10^b^
M32	Vehicle silhouettes	SGBP	72.64	73.82	9
		MRAN	59.36	59.78	102
		ELM	76.84	77.08	150
		SaE-ELM	77.73	78.23	120
		SVM	73.79	69.74	247^a^
		SAHRBF-BP	78.21	78.65	93&7^b^
M33	Waveform1	SGBP	53.26	60.39	11
		MRAN	83.36	82.28	371
		ELM	85.92	84.86	200
		SaE-ELM	86.71	86.24	100
		SVM	86.37	86.22	1854^a^
		SAHRBF-BP	87.53	87.13	326&9^b^

^a^Support vectors.

^b^RBF&BP hidden nodes.

**Table 9:** A few cases of failures in SAHRBF-BP compared with other learning algorithms on benchmark multi-class data sets.

Index	Data sets	Methods	Testing *η*_*o*_	Testing *η*_*a*_	*N*_*H*_ nodes
M02	Breast tissue	SGBP	54.21	53.49	6
		MRAN	54.51	53.25	12
		ELM	57.60	57.28	30
		SaE-ELM	58.33	57.76	20
		SVM	59.52	58.84	31^a^
		SAHRBF-BP	58.26	57.42	15&6^b^
M07	Glass	SGBP	58.43	57.87	7
		MRAN	63.79	62.61	31
		ELM	66.72	66.24	40
		SaE-ELM	67.26	66.82	20
		SVM	65.14	64.83	94^a^
		SAHRBF-BP	64.84	63.20	13&7^b^
M08	Hayes-Roth	SGBP	68.73	66.53	7
		MRAN	70.76	69.32	27
		ELM	71.43	70.27	40
		SaE-ELM	72.26	71.80	30
		SVM	74.12	74.58	68^a^
		SAHRBF-BP	72.68	71.42	12&5^b^
M19	Air	SGBP	72.14	74.63	8
		MRAN	85.59	84.51	42
		ELM	86.26	85.64	50
		SaE-ELM	87.03	87.23	30
		SVM	88.42	88.16	92^a^
		SAHRBF-BP	87.27	86.45	38&6^b^
M23	Forest	SGBP	45.26	44.52	8
		MRAN	66.25	64.71	73
		ELM	68.56	67.63	60
		SaE-ELM	68.84	68.38	40
		SVM	72.19	74.53	125^a^
		SAHRBF-BP	69.93	69.37	51&5^b^
M27	Libras	SGBP	42.69	–	9
		MRAN	50.53	–	96
		ELM	52.78	–	120
		SaE-ELM	52.26	–	80
		SVM	56.24	–	243^a^
		SAHRBF-BP	54.67	–	82&7^b^

^a^Support vectors.

^b^RBF&BP hidden nodes.

For Cardiotocography data set, the overall testing accuracy of SAHRBF-BP outperforms SVM by approximately 1.3%, and SaE-ELM, ELM, MRAN by approximately 2%-9.8%. The average testing accuracy of SAHRBF-BP outperforms SVM by approximately 0.8%, and SaE-ELM, ELM, MRAN by approximately 4%-9.7%.

For Knowledge data set, the overall testing accuracy of SAHRBF-BP outperforms SVM by approximately 2.5%, and SaE-ELM, ELM, MRAN by approximately 2.9%-4.9%. The average testing accuracy of SAHRBF-BP outperforms SVM by approximately 1%, and SaE-ELM, ELM, MRAN by approximately 3%-6.1%.

For Seeds data set, the overall testing accuracy of SAHRBF-BP outperforms SVM by approximately 1.6%, and SaE-ELM, ELM, MRAN by approximately 8.8%-11.7%.

For Wine data set, the overall testing accuracy of SAHRBF-BP outperforms SVM by approximately 1.2%, and SaE-ELM, ELM, MRAN by approximately 0.4%-8.4%.

For Yeast data set, the overall and average testing accuracies of SAHRBF-BP outperform SVM by approximately 2.5%, and SaE-ELM, ELM, MRAN by approximately 4.3%-6.3%.

For Image segmentation data set, the overall testing accuracy of SAHRBF-BP outperforms SVM by approximately 1.7%, and SaE-ELM, ELM, MRAN by approximately 1.1%-6.7%.

For Landsat data set, the overall testing accuracy of SAHRBF-BP outperforms SVM by approximately 0.7%, and SaE-ELM, ELM, MRAN by approximately 1.1%-4.7%. The average testing accuracy of SAHRBF-BP outperforms SVM by approximately 1%, and SaE-ELM, ELM, MRAN by approximately 1.4%-6.4%.

For Steel data set, the overall testing accuracy of SAHRBF-BP outperforms SVM by approximately 2.2%, and SaE-ELM, ELM, MRAN by approximately 0.9%-9.1%. The average testing accuracy of SAHRBF-BP outperforms SVM by approximately 2%, and SaE-ELM, ELM, MRAN by approximately 1.2%-10%.

For Turkie data set, the overall testing accuracy of SAHRBF-BP outperforms SVM by approximately 1.4%, and SaE-ELM, ELM, MRAN by approximately 2%-11%. The average testing accuracy of SAHRBF-BP outperforms SVM by approximately 1.4%, and SaE-ELM, ELM, MRAN by approximately 1.9%-10.9%.

For Vehicle silhouettes data set, the overall and average testing accuracies of SAHRBF-BP are clearly higher than MRAN. The overall testing accuracy outperforms SVM by approximately 4.4%, and SaE-ELM, ELM by approximately 0.5%-1.6%. The average testing accuracy of SAHRBF-BP outperforms SVM by approximately 8.9%, and SaE-ELM, ELM by approximately 0.4%-1.5%.

For Waveform1 data set, the overall testing accuracy of SAHRBF-BP outperforms SVM by approximately 1.2%, and SaE-ELM, ELM, MRAN by approximately 0.8%-4.2%. The average testing accuracy of SAHRBF-BP outperforms SVM by approximately 0.9%, and SaE-ELM, ELM, MRAN by approximately 0.9%-4.9%.

In Table 9, for Breast tissue data set, the overall and average testing accuracies of SAHRBF-BP are lower than SVM about 1.3% and 1.4%, respectively. For Glass data set, the overall testing accuracy of SAHRBF-BP is lower than ELM about 1.9%, SaE-ELM about 2.4%, and SVM about 0.3%. The average testing accuracy of SAHRBF-BP is lower than ELM, SaE-ELM and SVM about 3%, 3.6%, 1.6%, respectively. For Hayes-Roth data set, the overall and average testing accuracies of SAHRBF-BP are lower than SVM about 1.4% and 3.2%, respectively. For Air data set, the overall testing accuracy of SAHRBF-BP is lower than SVM about 1.2%. The average testing accuracy of SAHRBF-BP is lower than SVM about 1.7%, and SaE-ELM about 0.8%. For Forest data set, the overall and average testing accuracies of SAHRBF-BP are lower than SVM about 2.3% and 5.2%, respectively. For Libras data set, the overall testing accuracy of SAHRBF-BP is lower than SVM about 1.6%.

Similar to benchmark binary class data sets, the overall classification accuracy of SAHRBF-BP is higher than other learning algorithms on most multi-class low dimensional data sets, and the overall classification accuracy of SAHRBF-BP is decreased on a number of multi-class high dimensional data sets. However, for multi-class high dimensional data sets, when the number of training samples is sufficient, a relatively high classification accuracy of SAHRBF-BP can be obtained. The main reason is that enough training samples can offset the random distribution of sample space to a great extent. Under these circumstances, the methods of potential function clustering and heterogeneous samples repulsive force are still valid.

#### Benchmark large number of samples classification problems

**Fig 15:**
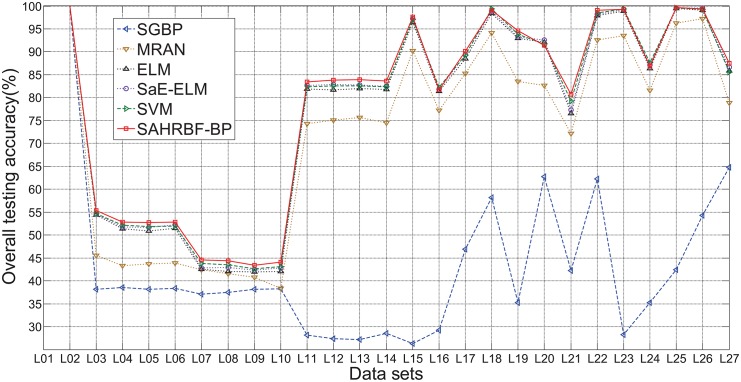
Overall testing accuracy comparisons between SAHRBF-BP and other algorithms on large number of samples data sets.

In this section, 27 large number of samples data sets are used to evaluate the performance of SAHRBF-BP. Fig 15 shows the overall testing accuracy comparisons between SAHRBF-BP and other learning algorithms. For large number of samples data sets, the overall testing accuracy of SAHRBF-BP is comparable with other learning algorithms on A1a(adult)(L01), A6a(adult)(L02) data sets, and SVM, SaE-ELM, ELM on Cod_rna(L15), Credit(L16), Gas(2013)(L18), Record(L23), Sensorless(L24), Skin(L25), Shuttle(L26) data sets. The overall testing accuracy of SAHRBF-BP is slightly lower than SaE-ELM, ELM on Letter(L20) data set. For other data sets, the overall testing accuracy of SAHRBF-BP is higher than other learning algorithms to varying degrees.

Table 10 gives performance comparisons between SAHRBF-BP and other learning algorithms on partial large number of samples data sets. The overall and average testing accuracies of SAHRBF-BP are clearly higher than SGBP on each large number of samples data set, except for A1a(adult) and A6a(adult) data sets. For Action2(normal)(L04) data set, the overall testing accuracy of SAHRBF-BP outperforms SVM by approximately 0.7%, and SaE-ELM, ELM, MRAN by approximately 1.1%-9.6%. For Action1(aggressive) data set, the overall testing accuracy of SAHRBF-BP outperforms SVM by approximately 0.8%, and SaE-ELM, ELM, MRAN by approximately 1.7%-2.1%. The average testing accuracy of SAHRBF-BP outperforms SVM by approximately 1.2%, and SaE-ELM, ELM, MRAN by approximately 1.8%-2.5%. For Action2(abnormal detection) data set, the overall testing accuracy of SAHRBF-BP outperforms SVM by approximately 1.2%, and SaE-ELM, ELM, MRAN by approximately 1%-8.7%. For Action3(abnormal detection) data set, the overall testing accuracy of SAHRBF-BP outperforms SVM by approximately 1.4%, and SaE-ELM, ELM, MRAN by approximately 1.2%-8.2%. The average testing accuracy of SAHRBF-BP outperforms SVM by approximately 0.9%, and SaE-ELM, ELM, MRAN by approximately 1%-8.2%. For Ijcnn1 data set, the overall testing accuracy of SAHRBF-BP outperforms SVM by approximately 0.7%, and SaE-ELM, ELM, MRAN by approximately 1.3%-11%. The average testing accuracy of SAHRBF-BP outperforms SVM by approximately 1.1%, and SaE-ELM, ELM, MRAN by approximately 2%-11.5%. For letter data set, the overall and average testing accuracies of SAHRBF-BP outperform SVM by approximately 0.5%, and MRAN by approximately 9.6% and 9.8%, respectively. However, the overall and average testing accuracy of SAHRBF-BP is lower than SaE-ELM about 0.4%, and ELM about 0.6% and 0.5%, respectively. For Occupancy data set, the overall testing accuracy of SAHRBF-BP outperforms SVM by approximately 1.2%, and SaE-ELM, ELM, MRAN by approximately 1%-8.7%. For Action3(abnormal detection) data set, the overall testing accuracy of SAHRBF-BP outperforms SVM by approximately 1.4%, and SaE-ELM, ELM, MRAN by approximately 2.2%-8.5%. The average testing accuracy of SAHRBF-BP outperforms SVM by approximately 1.5%, and SaE-ELM, ELM, MRAN by approximately 2.7%-8.6%.

**Table 10:** Performance comparisons between SAHRBF-BP and other learning algorithms on partial large number of samples data sets.

Index	Data sets	Methods	Testing *η*_*o*_	Testing *η*_*a*_	*N*_*H*_ nodes
L01	A1a(adult)	SGBP	100	100	8
		MRAN	100	100	76
		ELM	100	100	100
		SaE-ELM	100	100	50
		SVM	100	100	386^a^
		SAHRBF-BP	100	100	58&5^b^
L04	Action2(normal)	SGBP	38.52	–	40
		MRAN	43.29	–	2441
		ELM	51.36	–	4000
		SaE-ELM	51.74	–	3500
		SVM	52.12	–	5838^a^
		SAHRBF-BP	52.87	–	2123&20^b^
L07	Action1(aggressive)	SGBP	37.06	36.64	40
		MRAN	42.48	42.21	2549
		ELM	42.58	42.36	3500
		SaE-ELM	42.86	42.91	3000
		SVM	43.77	43.54	6256^a^
		SAHRBF-BP	44.61	44.73	2258&22^b^
L12	Action2(abnormal detection)	SGBP	28.17	–	40
		MRAN	75.12	–	3225
		ELM	81.72	–	4000
		SaE-ELM	82.83	–	3200
		SVM	82.56	–	5515^a^
		SAHRBF-BP	83.81	–	2432&24^b^
L13	Action3(abnormal detection)	SGBP	27.21	28.62	40
		MRAN	75.67	75.39	3173
		ELM	81.98	81.82	4000
		SaE-ELM	82.72	82.68	3200
		SVM	82.54	82.76	5584^a^
		SAHRBF-BP	83.92	83.64	2471&24^b^
L19	Ijcnn1	SGBP	35.28	47.62	40
		MRAN	83.57	82.25	1832
		ELM	92.93	90.82	3000
		SaE-ELM	93.26	91.78	2000
		SVM	93.81	92.61	3974^a^
		SAHRBF-BP	94.56	93.74	1657&18^b^
L20	Letter	SGBP	62.72	62.24	30
		MRAN	82.65	82.31	2630
		ELM	92.83	92.67	3000
		SaE-ELM	92.61	92.57	2000
		SVM	91.74	91.58	3668^a^
		SAHRBF-BP	92.26	92.12	2146&20^b^
L21	Occupancy	SGBP	42.26	40.83	30
		MRAN	72.14	71.62	1263
		ELM	76.52	75.18	1400
		SaE-ELM	78.30	77.38	1000
		SVM	79.24	78.66	1863^a^
		SAHRBF-BP	80.67	80.21	862&13^b^

^a^Support vectors.

^b^RBF&BP hidden nodes.

From Fig 15 and Table 10, we can see that for large number of samples data sets, the classification accuracy of SAHRBF-BP is higher than other learning algorithms in general. Enough training samples can effectively reflect the actual distribution of entire data sets, and the superiority of potential function clustering and heterogeneous samples repulsive force can be fully demonstrated.

### Discussion

#### Selection of the initial width parameters for SAHRBF-BP

The width parameters can be used to control the classification accuracy and generalization performance. To optimize the coverage of each class of samples, the center adjustment and the width adjustment strategy are combined together. When an initial width is given, for each generated RBF hidden node, the center is iteratively adjusted to a suitable position, and the width is then adjusted only once. To reduce the range of initial width values, we execute a preprocessing step for the sample space. For all benchmark classification problems, the inputs to each algorithm are scaled appropriately to fall between -1 and +1.

In addition, the initial width *σ* and the minimum width *σ*_min_ are related to each other. According to Eq (16), the adjusted width is in the range between *σ*_min_ and *σ*; that is, $\sigma_{m}\in \left[\sigma_{\min},\sigma \right]$, where *σ*_min_ ∈ {*σ*_min_|*σ* − *ϑ* ≤ *σ*_min_ < *σ*, *σ*_min_ > 0}. To guarantee the generalization performance, here we set *ϑ* = 0.2. Thus, when the initial width *σ* is given, the minimum width *σ*_min_ can be determined accordingly. For example, if *σ* = 0.5, *σ*_min_ can be selected in the set {*σ*_min_|*σ* − 0.2 ≤ *σ*_min_ < *σ*}. To simplify this case, *σ*_min_ can be selected in the set {0.3, 0.4}, and the one with the lowest validation error is selected as the suitable minimum width.

#### Effect of the initial width parameters on SAHRBF-BP

**Fig 16:**
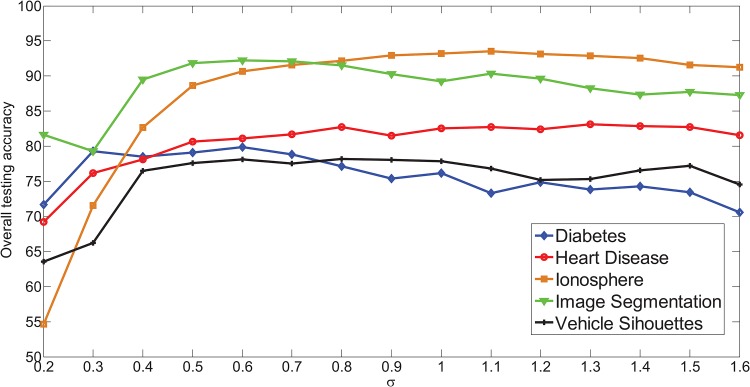
Effect of initial width parameters on SAHRBF-BP. (A) *σ*- Overall testing accuracy (B) *σ*- Number of RBF hidden nodes.

When the initial width changes, the number of generated RBF hidden nodes and node parameters will change accordingly. Here Diabetes, Heart disease, Ionosphere, Image segmentation and Vehicle silhouettes data sets are used to evaluate the effect of the initial width parameters on SAHRBF-BP. In Fig 16, when the initial width value is too small, the overall classification accuracy is poor and the network size for the RBF hidden layer is large, e.g., for Heart disease data set, when *σ* = 0.2, the number of generated RBF hidden neurons is 151 and is equal to the number of training samples.

This result demonstrates the corresponding RBF hidden nodes will be established at each training sample, and the generated RBF hidden neurons will not cover other samples, thus the methods of potential function clustering and heterogeneous sample repulsive force are invalid and the overall classifying accuracy is poor in this case.

Thus, in SAHRBF-BP, to complete the effective coverage of the training sample space, an effective initial width parameter should be provided, which can generate proper RBF hidden neurons to cover the sample space. Note that the number of generated RBF hidden neurons should not be close to the number of the training samples, otherwise, SAHRBF-BP is invalid.

When the value of the initial width falls within a suitable range, the number of generated RBF hidden nodes will change, but a relatively stable classification accuracy can be achieved. For instance, for Image segmentation data set, when the range of initial widths is between 0.5 and 0.9, the overall testing accuracy ranges from 91.54% to 92.23%. Once initial width parameters are given, SAHRBF-BP can learn the sample space automatically and generate different RBF hidden nodes to adapt the sample space. Thus, SAHRBF-BP can counteract the effect of the initial width parameters to some extent.

#### Effect of the number of BP hidden nodes on SAHRBF-BP

In SAHRBF-BP, the nonlinear SGBP algorithm is used to adjust the weights of the BP network component, which further improves the classification result. However, this method results in an increase in the number of parameters to be selected, particularly the selection of the number of BP hidden nodes. For this problem, we conduct experiments on five UCI data sets and discuss the results.

**Fig 17:**
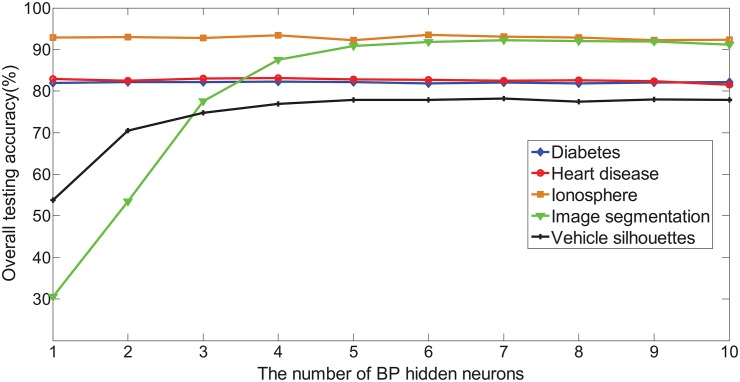
Effect of the number of BP hidden nodes on SAHRBF-BP.

Fig 17 shows the effect of the number of BP hidden nodes on SAHRBF-BP. The results show that for binary class classification problems, such as Diabetes, Heart disease and Ionosphere data sets, when the number of BP hidden nodes ranges from 1 to 10, a relatively stable classification accuracy can be achieved.

For multi-class classification problems, such as Image segmentation and Vehicle silhouettes data sets, when the number of BP hidden nodes is greater than 4, the overall classification accuracy also does not change considerably. Thus, the dependence on the number of BP hidden nodes is reduced.

For the SAHRBF-BP classifier, the adaptively mapping results for the RBF hidden nodes are processed and used for the input of BP network component, which improves the stability of the BP network component and effectively avoids falling into local minima for the BP algorithm. When the sample set is more complex, the momentum term can be used to improve the BP algorithm further.

#### Limitations for SAHRBF-BP

Compared with other training SLFNs algorithms, SAHRBF-BP shows excellent classification performance on artificial and most benchmark data sets. However, there are still some limitations for SAHRBF-BP.

For complex classification problems, to achieve good classification results, the number of training samples should not be too small. Otherwise, the randomness of training samples in the sample space is enhanced, which can not effectively reflect the actual distribution of entire data sets, especially for high dimensional data sets, and will lead to the methods of potential function clustering and heterogeneous sample repulsive force some extent of failure.

In addition, to ensure the effectiveness of learning, the initial kernel width should not be too small, which is another limitation for SAHRBF-BP. Otherwise, the generalization performance of the classifier will be greatly reduced, and each generated RBF hidden node does not cover the heterogeneous samples, which leads to the failure of heterogeneous sample repulsive force.

## Conclusion

In this paper, a structure-adaptive hybrid RBF-BP (SAHRBF-BP) classifier with an optimized learning strategy is presented. SAHRBF-BP is composed of a structure-adaptive RBF network and a BP network of cascade, where the number of RBF hidden nodes is adjusted adaptively according to the distribution of sample space. SAHRBF-BP makes use the global information of each class of training samples to generate the initial RBF hidden nodes, and then makes full use of the neighborhood information of each hidden node to optimize the hidden node parameters. Thus, SAHRBF-BP solves the problem of dimension change from sample space mapping onto feature space. In addition, it also effectively combines the stability of a RBF network and the generalization ability of a BP network to improve the classification performance. In this way, SAHRBF-BP simplifies the selection of the number of nodes in the BP hidden layer while further reducing the dependence on space mapping in the RBF hidden layer. The optimized learning strategy can generate RBF hidden nodes incrementally, as well as adjust the centers and width adaptively. The combination of the potential function clustering with heterogeneous sample repulsive force improves the classification accuracy of each hidden node; at the same time, it ensures a compact network size for the RBF hidden layer.

The performance of SAHRBF-BP is compared with that of other training SLFNs algorithms, namely SGBP, KMRBF, KMRBF-BP, MRAN, GAP-RBF, SVM, ELM, and SaE-ELM on different data sets. In each training SLFNs algorithm, SVM is still the most stable classifier. Compared to other algorithms, the classification performance of SVM is maintained at a relatively high level on each data set in general. Overall, for high dimensional with too small training samples data sets, the classification performance of SVM outperforms SAHRBF-BP clearly. However, with the increase of the number of samples in data sets, the randomness of training samples in the sample space is gradually being eliminated. On the basis of effective learning of sample space, SAHRBF-BP shows its unique advantages. On most low dimensional and large number of data sets, the results show the classification performance of SAHRBF-BP outperforms other training SLFNs algorithms.

In the future, we will focus on imbalanced data classification problems. For imbalanced data classification problems, the samples of minority classes and the samples in boundary regions should be emphasized more, which contain more classification information, thus how to measure and select these samples is particularly important. Further studies are needed to address these concerns.
